# Genome-wide methylation and expression analyses reveal the epigenetic landscape of immune-related diseases for tobacco smoking

**DOI:** 10.1186/s13148-021-01208-0

**Published:** 2021-12-09

**Authors:** Ying Mao, Peng Huang, Yan Wang, Maiqiu Wang, Ming D. Li, Zhongli Yang

**Affiliations:** 1 State Key Laboratory for Diagnosis and Treatment of Infectious Diseases, National Clinical Research Center for Infectious Diseases, Collaborative Innovation Center for Diagnosis and Treatment of Infectious Diseases, The First Affiliated Hospital, Zhejiang University School of Medicine, Hangzhou, China; 2 Research Center for Air Pollution and Health, Zhejiang University, Hangzhou, China

**Keywords:** Tobacco smoking, Epigenetics, WGBS, DMR, FLT1/VEGFR1, RNA-seq

## Abstract

**Background:**

Smoking is a major causal risk factor for lung cancer, chronic obstructive pulmonary disease (COPD), cardiovascular disease (CVD), and is the main preventable cause of deaths in the world. The components of cigarette smoke are involved in immune and inflammatory processes, which may increase the prevalence of cigarette smoke-related diseases. However, the underlying molecular mechanisms linking smoking and diseases have not been well explored. This study was aimed to depict a global map of DNA methylation and gene expression changes induced by tobacco smoking and to explore the molecular mechanisms between smoking and human diseases through whole-genome bisulfite sequencing (WGBS) and RNA-sequencing (RNA-seq).

**Results:**

We performed WGBS on 72 samples (36 smokers and 36 nonsmokers) and RNA-seq on 75 samples (38 smokers and 37 nonsmokers), and cytokine immunoassay on plasma from 22 males (9 smokers and 13 nonsmokers) who were recruited from the city of Jincheng in China. By comparing the data of the two groups, we discovered a genome-wide methylation landscape of differentially methylated regions (DMRs) associated with smoking. Functional enrichment analyses revealed that both smoking-related hyper-DMR genes (DMGs) and hypo-DMGs were related to synapse-related pathways, whereas the hypo-DMGs were specifically related to cancer and addiction. The differentially expressed genes (DEGs) revealed by RNA-seq analysis were significantly enriched in the “immunosuppression” pathway. Correlation analysis of DMRs with their corresponding gene expression showed that genes affected by tobacco smoking were mostly related to immune system diseases. Finally, by comparing cytokine concentrations between smokers and nonsmokers, we found that vascular endothelial growth factor (VEGF) was significantly upregulated in smokers.

**Conclusions:**

In sum, we found that smoking-induced DMRs have different distribution patterns in hypermethylated and hypomethylated areas between smokers and nonsmokers. We further identified and verified smoking-related DMGs and DEGs through multi-omics integration analysis of DNA methylome and transcriptome data. These findings provide us a comprehensive genomic map of the molecular changes induced by smoking which would enhance our understanding of the harms of smoking and its relationship with diseases.

**Supplementary Information:**

The online version contains supplementary material available at 10.1186/s13148-021-01208-0.

## Background

Tobacco smoking is a major causal risk factor for various diseases, including cancers, respiratory problems, cardiovascular disorders, and others [1]. There are more than one billion tobacco users in the world, about 1/3 of them in China [2]. Although smoking cessation campaigns and legislative actions led to a reduction of 6 million in the number of tobacco users, smoking is still a leading preventable cause of death, killing more than 8 million people each year, most of them in developing countries [2].

More than 4,000 compounds have been identified in the particulate and vapor phase of tobacco, which include about 60 known carcinogens, such as nitrosamines, polycyclic aromatic hydrocarbons, and aromatic amines [3]. Some of these components are reported to involve in innate and adaptive immune responses and inflammatory processes, thereby increasing the prevalence of smoking-related diseases such as COPD and lung cancer [4, 5]. Cigarette smoke induces alveolar macrophages (AMs) to express more lysosomal enzymes and secrete elastase, which may damage the connective tissue and parenchymal cells of the lung and may play a key role in chronic bronchitis and emphysema effect [5, 6]. It is known that one-third of cancers can be attributed to smoking, especially lung cancer, oral cancer, pancreatic cancer, esophageal cancer, and kidney cancer [7]. Lung cancer is the most frequent one in the world and the leading cause of cancer deaths  [8]. However, the underlying molecular mechanisms linking smoking and related diseases have not been well explored, especially at the level of epigenomics that is greatly influenced by human living environment.

DNA methylation (DNAm) is a highly dynamic epigenetic change that attaches methyl groups to nucleotides and is one of major mechanisms underlying the effects of tobacco smoking [9]. Studies based on Illumina 450 K and 850 K methylation arrays have shown that numerous CpG sites are significantly associated with smoking [10–15]. The hypomethylated genes probably as a result of tobacco smoking are linked to immune diseases, lung cancer, and death [14, 16, 17]. At the level of the transcriptome, RNA expression changes are commonly quantified using microarrays with peripheral whole blood and alveolar macrophages. Recent studies have reported that DNAm that is abnormal in the regulatory elements modulates the expression of smoking susceptibility genes and is associated with a higher risk of various cancers [11, 18–20]. However, array-based targeting gene studies, whether at the level of epigenomics or the transcriptome, can reveal only limited molecular changes induced by tobacco smoking.

**Fig. 1:**
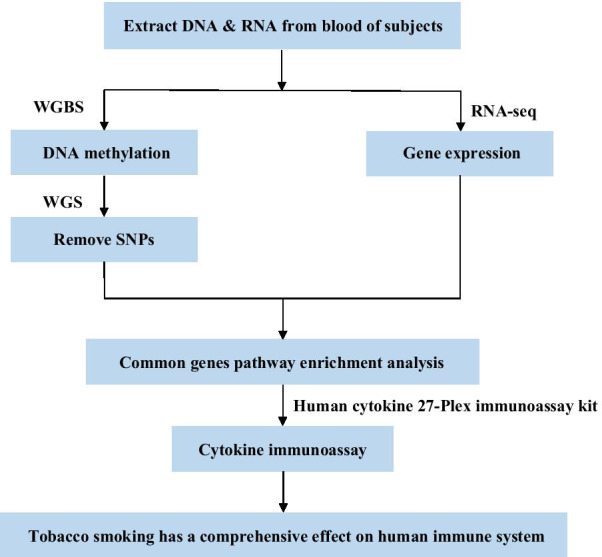
Discovery strategy. To overcome the limitation of commonly used in DNA methylation arrays, which capture only a limited number of CpG sites throughout the whole genome, we performed whole genome bisulfite sequencing (WGBS) on 72 males in the city of Jincheng in China. Rare variants (MAF < 0.05) were removed according to our whole genome sequencing (WGS) dataset. We also performed RNA sequencing analysis (RNA-seq) on 75 local male individuals with the goal of finding differentially expressed genes between smokers and nonsmokers at the genomic level

With the emergence of high-throughput next-generation sequencing, whole-genome bisulfite sequencing (WGBS) has greatly enriched our understanding of changes in methylation across the genome [21, 22]. RNA sequencing (RNA-seq) can interrogate not only existing annotated transcripts, but also new sequences and splice variants; and it can achieve a much higher resolution with low limits compared with standard whole-genome microarrays [23]. To the best of our knowledge, there have been few studies on the systematic analysis of the molecular effect of tobacco smoking on human beings by integrating DNA methylation and RNA-seq data at the genome level in Chinese samples [24]. By analyzing WGBS and RNA-seq data in both smokers and nonsmokers, in this study, we intended to: (1) depict the effects of tobacco exposure on genome-wide DNA methylation changes in Chinese adult male smokers; (2) explore the relations between smoking-related methylation and the corresponding RNA expression; and (3) integrate results from DNA methylation, RNA expression, and cytokine concentrations with the goal of revealing the molecular mechanism underlying the effects of tobacco smoking on smokers at multiple levels and exploring the relationship between smoking and related diseases (Fig. 1).

## Results

### Correlation of tobacco smoking with DNA methylation changes throughout the whole genome

After WGBS analysis of all samples, we obtained an average of about 700 million (± 75 million; SD) paired-end reads of 150 bp for each sample. Table 1 lists the detailed demographic characteristics of the samples included in the study. For the captured 25 million autosomal CpG sites from each of 72 subjects, we acquired the single base resolution methylation ratio with an average coverage of 88.21% (± 1.19%; SD) and conversion rate of 79.31% (± 3.25%; SD). After removal of rare SNPs with MAF < 0.05, a total of 24,479,261 CpG sites with an average depth of 12.53 × (± 1.45; SD) were included in the study.

**Table 1:** Sample characteristics

Characteristics	WGBS		RNA-seq	
	Smokers	Nonsmokers	Smokers	Nonsmokers
Sample size	36	36	38	37
Average age (SD)	41.0 (2.08)	41.3 (2.51)	42.84 (5.55)	42.86 (5.48)
BMI (SD)	24.3 (3.33)	25.1 (3.55)	24.17 (2.75)	25.89 (3.03)
CPD (SD)	21.58 (3.33)	0	23.55 (8.70)	0
FTND (SD)	5.42 (2.12)	0	6.34 (1.61)	0
Miner (Non-miners)	18 (18)	18 (18)	29 (9)	21 (16)

*BMI* Body Mass Index, *CPD*, cigarettes per day, *FTND* Fagerstrom test for nicotine dependence

**Fig. 2:**
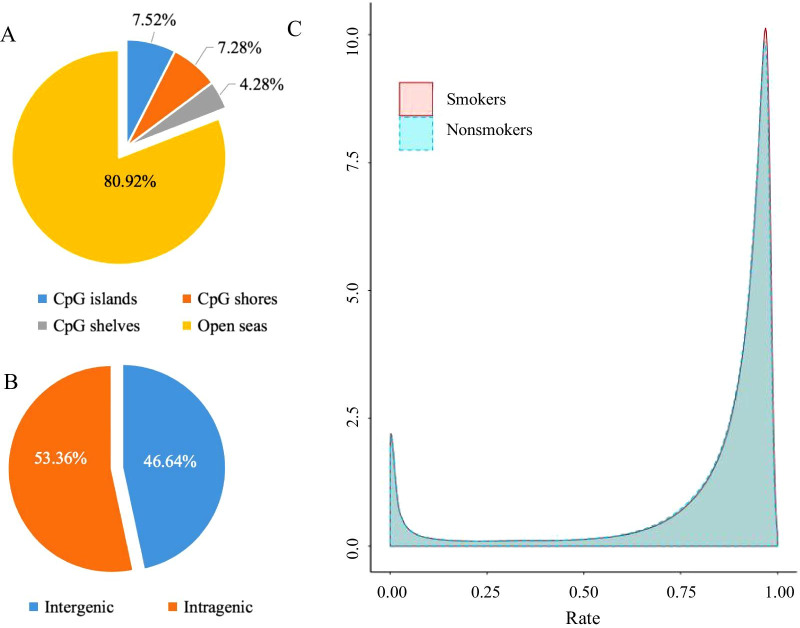
Genome distribution of CpGs captured by WGBS. The distributions of CpGs summarized based on CpG densities (**A**) and genomic locations (**B**). **C** Distribution of methylation level density of each site in the whole genome. *Note*: X = degree of methylation; Y = the CpG site density corresponding to the level of methylation; WGBS = whole genome bisulfite sequencing

About 20% of the CpG sites were located in CpG-rich areas, while the rest mapped to regions with lower CpG density (Fig. 2A). Relative to genomic background, more than half of the total CpGs were annotated to genes based on Human Genome Reference GRCH37, and the rest were located in intergenic regions (Fig. 2B).

**Fig. 3:**
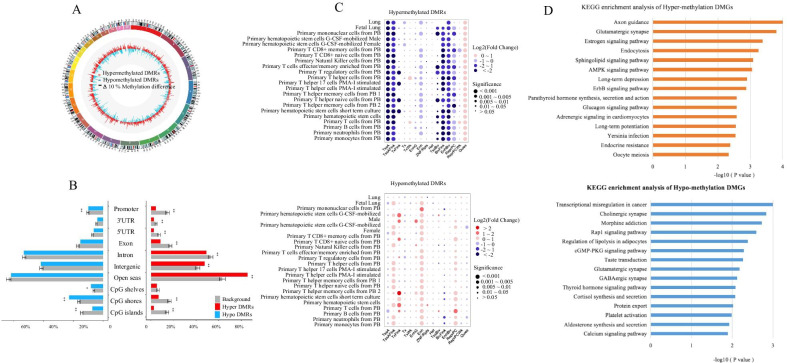
Distribution of SM-DMRs across the whole genome. **A** Tobacco smoking is associated with DNA methylation changes across the whole genome. Circles represent DNA methylation levels for hypermethylation (outer circle, red) and hypomethylation (inner circle, blue). The height of each bar indicates the methylation change between smokers and nonsmokers. **B** The distribution ratio and enrichment results of DMRs summarized based on genomic locations. Red and blue bars in the figure represent the SM-DMRs and each grey bar is the average proportion of the 1000 groups of control regions for each genome feature. The significance level and standard deviation are shown in the figure (*P < 0.05, **P < 0.01). **C** SM-DMRs enrichment results of epigenomic annotations in different cell types and tissues based on Roadmap Epigenomics Project. A node represents enrichment results of SM-DMRs in a certain chromatin state in a specific cell type or tissue. The node color refers to the direction of enrichment (log_2_Fold Change), and the size indicates the level of significance. Empirical *P* value was generated by simulating null distribution of 1000 groups of control areas in the genome. **D** KEGG pathway enrichment analysis of hypermethylated and hypomethylated DMGs (Top 15 pathways). *SM* smoking associated, *DMR* differentially methylated region, *DMGs* differentially methylated genes, *TssA* Active TSS, *TssAFlnk* Flanking Active TSS, *TxFlnk* Transcr.at gene 5' and 3', *Tx* Strong transcription, *TxWk* Weak transcription, *EnhG* Genic enhancers, *Enh* Enhancers, *ZNF/Rpts* ZNF genes & repeats, *Het* Heterochromatin, *TssBiv* Bivalent/Poised TSS, *BivFlnk* Flanking Bivalent TSS/Enh, *EnhBiv* Bivalent Enhancer, *ReprPC* Repressed PolyComb, *ReprPCWk* Weak Repressed PolyComb, *Quies* Quiescent/Low

The raw methylation status distribution of genome-wide CpG sites revealed the presence of two obvious peaks (Fig. 2C), with the one concentrated in low methylation sites (0–0.1) and the other in high methylation sites (0.9–1.0). In addition, the landscape of 2,440 smoking-associated DMRs at a significance level of Stouffer–Liptak *P* value of ≤ 1.00 × 10^–4^ was identified, with an average methylation difference of 3.4% (0.3 ~ 20%) in these regions, among which 74.59% were highly methylated and 25.41% were low methylated compared with nonsmokers. The ratio between DMRs in each direction was approximately 3:1. Figure 3A shows the overall effect of tobacco smoking on methylation in the whole genome.

### DMR enrichment analysis

Figure 3B shows that hypermethylated DMRs were significantly depleted in the promoter regions, 5′-UTRs, 3′-UTRs, exons, introns, CpG shores, and islands and were enriched in intergenic regions and open seas compared with background regions. Hypomethylated DMRs were significantly enriched in the CpG shores and CpG shelves and depleted in promoter regions and CpG islands compared with background regions. From the results of chromatin state enrichment analysis, we found that hypermethylated smoking-related DMRs were frequently depleted in the active TSS, flanking active TSS, and bivalent enhancer but were significantly enriched in quiescent/low areas (defined as inactive chromatin states with closed chromatin, very low signals for all available histone marks and varying levels of DNA methylation), whereas hypomethylated SM-DMRs were significantly enriched in predicted enhancers areas based on the data from 20 blood cell types and two lung tissues (Fig. 3C).

### Biological functions of DMR-related genes

We found a total of 1205 hypermethylated genes and 534 hypomethylated genes through the gene annotation of DMRs (see Additional file 1: Table S4). To determine the biological functions of these genes, we performed Kyoto Encyclopedia of Genes and Genomes (KEGG) enrichment analysis (Fig. 3D, Additional file 1: Tables S5 and S6). The top three pathways of hypermethylated genes were axon guidance (has04360; *P* = 1.72 × 10^–5^), glutamatergic synapse (hsa04724; *P* = 1.56 × 10^–4^), and estrogen signaling pathway (hsa04915; *P* = 4.18 × 10^–4^), respectively. On the other hand, we identified 30 pathways for hypomethylated genes (*P* value < 0.05), with the first one being cancer-related pathways, the second synapse-related pathways (i.e., cholinergic synapse), and the third related to morphine addiction.

### Identification of differentially expressed genes (DEGs)

**Fig. 4:**
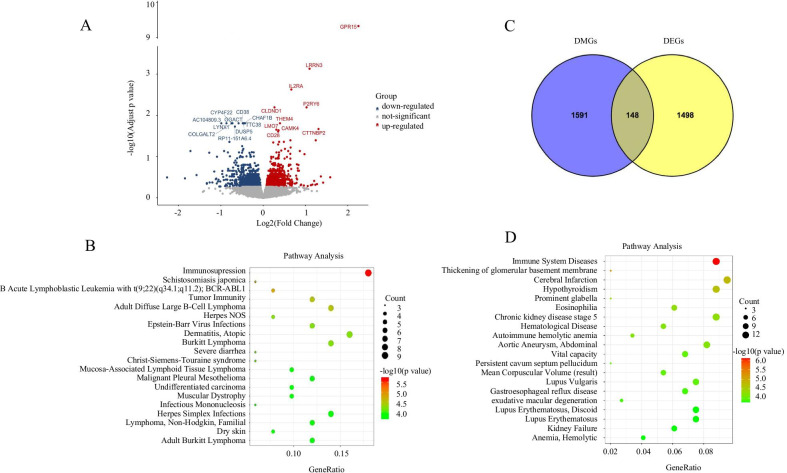
Genes detected in both methylation and RNA expression. **A** Volcano plot shows the differentially expressed genes between smokers and nonsmokers. Each dot corresponds to a gene and color corresponds to the direction. **B** Pathway enrichment analysis of DEGs by DisGeNET. **C** Overlapping genes between DMGs and DEGs. **D** Pathway enrichment analysis of overlapping genes in methylation and mRNA datasets by DisGeNET. The 20 most significantly enriched pathways are illustrated in dot plots. Gene ratio refers to the proportion of DEGs belonging to a specific term. Node size (count) refers to the number of DEGs within each term and the color indicates the level of significance (− log_10_ *P* value). *DMGs* differentially methylated genes, *DEGs* differentially expressed genes

Our RNA-Seq analysis revealed 18,651 transcripts, of which 17,566 were annotated to the human genome, with 13,838 protein-coding genes, 695 lincRNAs, and 1,348 pseudogenes and other RNAs. Statistical analysis revealed 55 DEGs between smokers and nonsmokers after correction for multiple testing (Fig. 4A; see Additional file 1: Table S7, for details), which included 51 protein-coding RNAs, two long non-coding RNAs (i.e., AC009299.3 and FAM225B), one sense intronic, and one pseudogene. The top two protein-coding genes were *GPR15* (log_2_FC = 2.25; *P* = 2.54 × 10^–14^) and *LRRN3* (log_2_FC = 1.10; *P* = 7.92 × 10^–8^).

In addition, we analyzed gene–disease associations using the DisGeNET database (Fig. 4B). The results showed that the most significant gene–disease sets were significantly enriched in “immunosuppression” (GO: C4048329; *P* = 2.00 × 10^–6^).

### Correlation analysis of DMRs’ methylation and gene expression

Next, we determined whether genes differentially expressed in smokers and nonsmokers were correlated with smoking-induced epigenetic changes. We analyzed correlations between 1,746 significant DMGs (See Additional file 1: Table S8) and 1,646 significant DEGs (see Additional file 1: Table S9) and found 148 overlapping genes between the DMG and DEG sets (defined as DMR–DEG pairs; Fig. 4C; see Additional file 1: Table S10). These 148 common genes were classified into four categories according to the directions of DNA methylation and gene expression relative to nonsmokers: 34 “Hyper-Up” for the hypermethylated and upregulated genes; 57 “Hyper-Down” for the hypermethylated and downregulated genes; 26 “Hypo-Up” for the hypomethylated and upregulated genes and 31 “Hypo-Down” for the hypomethylated and downregulated genes (Table 3). Pathway analysis showed that these 148 smoking-related genes were enriched primarily in immune system diseases (*P* = 7.94 × 10^–7^; Table 2; Fig. 4D). We also performed KEGG pathway enrichment analysis and obtained similar results (see Additional file 1: Table S11, for details).

**Table 2:** A list of top 20 pathways revealed by pathway enrichment analysis of common genes in both DNA methylation and RNA-Seq datasets by using Metascape

GO term	Description	*P* value
C0021053	Immune system diseases	7.94E−07
C0445347	Thickening of glomerular basement membrane	5.01E−06
C0007785	Cerebral infarction	1.58E−05
C0020676	Hypothyroidism	2.00E−05
C1860247	Prominent glabella	5.01E−05
C2316810	Chronic kidney disease stage 5	5.01E−05
C0014457	Eosinophilia	5.01E−05
C0018939	Hematological disease	6.31E−05
C0162871	Aortic aneurysm, abdominal	6.31E−05
C0002880	Autoimmune hemolytic anemia	6.31E−05
C0524587	Mean corpuscular volume (result)	1.00E−04
C1840380	Persistent cavum septum pellucidum	1.00E−04
C0042834	Vital capacity	1.00E−04
C0024131	Lupus vulgaris	1.00E−04
C0017168	Gastroesophageal reflux disease	1.26E−04
C2237660	Exudative macular degeneration	1.26E−04
C0024138	Lupus erythematosus, discoid	1.58E−04
C0002878	Anemia, hemolytic	1.58E−04
C0035078	Kidney failure	1.58E−04
C0409974	Lupus erythematosus	1.58E−04

Considering that hypermethylated DMRs were significantly depleted in the promoter regions, and the hypomethylated DMRs were enriched in the enhancer regions, we decided to focus on the DMRs located in genomic regulatory elements and performed correlation analysis between significant DMR methylation and their predicted target gene expression.

**Fig. 5:**
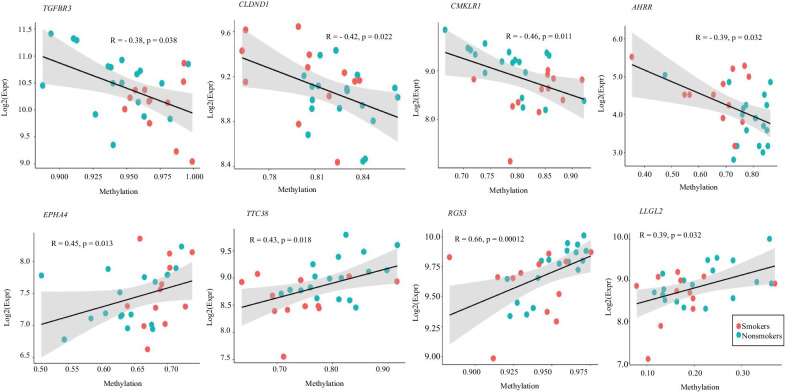
Correlation analysis between methylation and expression level of each target gene. Each dot corresponds to a sample and color corresponds to each group. The straight line represents the fitted curve, *R* and *P* value represents the Spearman correlation coefficient and *P* value respectively

Of the 196 DMRs located in the promoter regions, only chemerin chemokine-like receptor 1 (CMKLR1) showed a significant negative correlation between its hypermethylation and down-regulation of gene expression (Table 3). Among the 254 DMRs located in the enhancer regions (defined as differentially methylated enhancers; DMEs), correlation analysis showed that 22 enhancer-associated DMRs correlated significantly with target gene expression (Table 3), with 70% of them negatively correlated. These DMEs were divided into two categories, one acting on its host genes, and the other “commuting enhancers,” which was located in a gene but acted on other distal gene(s). These findings indicated that smoking-related differential methylation sites target mainly enhancers other than promoters. About 1% (22/254) of all differentially methylated regions in enhancers had a significant correlation with the expression of target genes (Fig. 5).

**Table 3:** Methylation and expression correlation analysis

Regulatory element	DMR			DEG		Correlation analysis
	Region	Genehancer ID	Direction (smokers–nonsmokers)	Target gene	Direction (smokers–nonsmokers)	*P* value
Promoter	chr12:108733790–108734067	GH12J108336	Hyper	*CMKLR1*	Down	0.0108
Promoter/enhancer	chr2:222312663–222313620	GH02J221447	Hyper	*EPHA4*	Up	0.0130
	chr2:74375246–74375595	GH02J074146	Hyper	*AUP1*	Down	0.0433
	chr12:108733790–108734067	GH12J108336	Hyper	*CMKLR1*	Down	0.0108
	chr12:48289327–48289770	GH12J047896	Hyper	*DDX23*	Down	0.0122
	chr11:126030937–126031289	GH11J126157	Hyper	*FAM118B*	Down	0.0181
	chr2:74375246–74375595	GH02J074146	Hyper	*HTRA2*	Down	0.0433
	chr22:22128415–22128928	GH22J021772	Hyper	*IGLV5-52*	Down	0.0424
	chr19:30158120–30158513	GH19J029660	Hyper	*PLEKHF1*	Down	0.0241
	chr11:116699588–116699946	GH11J116825	Hyper	*SIDT2*	Down	0.0159
	chr1:161513847–161514197	GH01J161539	Hyper	*SLAMF7*	Down	0.0324
	chr11:116699588–116699946	GH11J116825	Hyper	*TAGLN*	Down	0.0116
	chr1:92291397–92291719	GH01J091825	Hyper	*TGFBR3*	Down	0.0376
	chr5:372991–373651	GH05J000367	Hypo	*AHRR*	Up	0.0324
	chr3:98239609–98240661	GH03J098521	Hypo	*CLDND1*	Up	0.0220
	chr3:169757128–169757597	GH03J170037	Hypo	*GPR160*	Up	0.0131
	chr19:19928614–19928997	GH19J019816	Hypo	*ZNF506*	Up	0.0348
	chr19:38186831–38187042	GH19J037695	Hypo	*ZNF793*	Up	0.0194
	chr17:73684198–73684529	GH17J075683	Hypo	*LLGL2*	Down	0.0316
	chr11:67024748–67025170	GH11J067257	Hypo	*RAD9A*	Down	0.0420
	chr9:116164296–116164796	GH09J113396	Hypo	*RGS3*	Down	0.0001
	chr22:46442102–46442610	GH22J046040	Hypo	*TTC38*	Down	0.0179
	chr7:148723197–148723699	GH07J149026	Hypo	*ZNF282*	Down	0.0220

### Association between circulating cytokines and tobacco smoking

The above-mentioned results indicated that overlapping genes were significantly enriched in immune system-related diseases. Thus, we analyzed differences in 27 cytokines between smokers and nonsmokers. After comparing cytokine concentrations in the two groups, we found that vascular endothelial growth factor (VEGF; *P* = 0.03; 95% confidence interval [CI] 0.02–0.50) and FGF basic (*P* = 0.04; 95% CI − 0.01 to 0.34) level were significantly upregulated in smokers compared with nonsmokers (see Additional file 1: Table S12).

### Relation between smoking-associated hypomethylation and up-regulated expression of *FLT1*

**Fig. 6:**
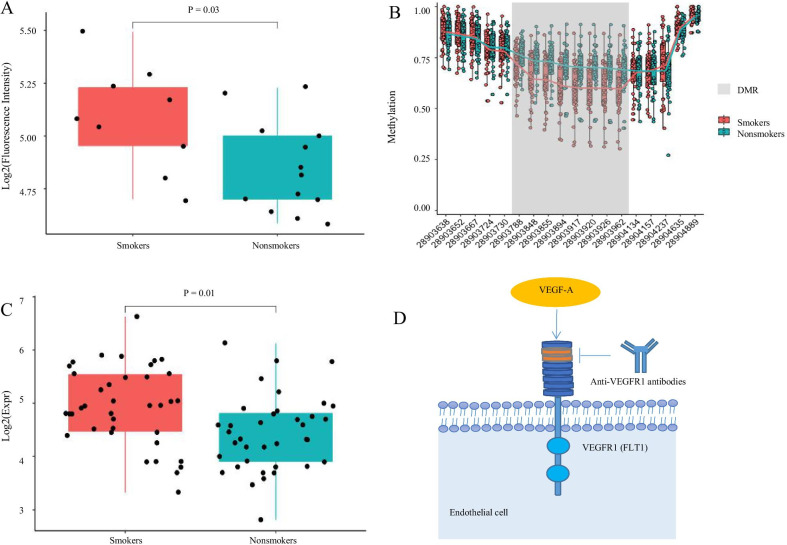
Differences on *VEGF* and *VEGFR1* between smokers and nonsmokers. Comparison of VEGF cytokine level (**A**) and *FLT1/VEGFR1* RNA expression (**C**) level between smokers and nonsmokers. Methylation level in *FLT1/VEGFR1* between smokers and nonsmokers (**B**). VEGF and its receptors (**D**). Each dot corresponds to a sample and color corresponds to each group. *VEGF* vascular endothelial growth factor, *VEGFR1* vascular endothelial growth factor receptor 1

The cytokine results showed that VEGF was significantly increased in smokers. We also detected DNA methylation and RNA of VEGF but failed to find any significant differences in them. However, we found that its receptor, FLT1/VEGFR1, was hypomethylated in its enhancer region (Δ methylation = 9.2%; *P* = 7.02 × 10^–5^) and significantly up-regulated in smokers (FC = 1.39; *P* = 8.7 × 10^–3^) (Fig. 6).

## Discussion

Smoking causes changes in human immune function and increases the risk of various cancers, but there are still few studies that can explain the molecular mechanism of the relationship between the two. In this study, we carried out genome-wide DNA methylation, RNA, and human cytokine analysis with the goal of finding molecular changes in blood linked to tobacco smoking exposure. We conclude that tobacco smoke has a significant effect on epigenetic modification and transcription regulation and is significantly associated with human immune system diseases, cancers, and synapse-related pathways.

Regular smokers have a lifetime risk of smoking-related cancer as high as 25%. Low methylation of specific CpG sites in *AHRR* (aryl-hydrocarbon receptor repressor) and *F2RL3* (factor II receptor-like 3) has been associated with an increased risk of chronic diseases and lung cancer, as well as with the total mortality rate [11, 16, 19, 25], and could be used as biological markers for tobacco smoking [26, 27]. We replicated the low methylation on these two genes (*AHRR*, *P* = 4.194 × 10^–7^; *F2RL3*, *P* = 3.34 × 10^–6^) in Chinese male smokers. By examining the genes identified in the gene expression response to methylation changes (Table 3), we also identified a number of new smoking-related important genes. *PLEKHF1* (Pleckstrin Homology And FYVE Domain Containing 1, chr19:30158120–30158513, Δ methylation = 8.02%, *P* = 6.91E-06), a gene was reported to induce apoptosis through the lysosomal–mitochondrial pathway [28]; *SLAMF7* (SLAM Family Member 7, chr1:161513847–161514197, Δ methylation = 5.84%, *P* = 6.85E-05), a self-ligand and has both activating and inhibitory functions in natural killer cells, which involves in the regulation and interconnection of innate and adaptive immune responses [29]. In addition, the hypomethylation on *JAG2* gene (chr14:105619745–105620094, Δ methylation = 10.05%, *P* = 4.23E-07) deserves our attention as well. It is a key ligand of the Notch signaling pathway and involved in the respiratory system process [30]. However, whether these methylation changes caused by smoking could be detected in Chinese female smokers remains to be investigated.

Compared with other studies, this research had the following strengths [24, 31, 32]. Firstly, the male subjects in our study came from the same area (Jincheng, Shanxi) and were mainly engaged in coal mining-related industry. Therefore, their living styles were relatively more uniform, which made the study population less variable and easy to control other potential variables. Second, our larger sample in WGBS (whole-genome bisulfite sequencing) on smoking was able to reveal more smoking-associated CpG loci at single-base resolution throughout the whole genome [21]. In spite of its higher cost, WGBS is capable of capturing significantly more human CpG sites (~ 90%) and providing true methylation amounts with a higher resolution relative to the commonly used array approach. Other than TSS areas, we acquired a more comprehensive DNA methylation map of gene body and intergenic areas. Our chromatin state annotation indicated that hypermethylated DMRs were depleted primarily in TSS, whereas hypomethylated DMRs were enriched in flanking regions of active TSS and enhancers. DNA methylation in the promoter region generally suppresses transcription or acts as a marker of a silenced gene [33, 34] because of hard binding of transcription factors or recruitment of transcription repressors [35, 36]. As for DNA methylation in the gene body region, the situation appears to be complex, although it was reported that there exists a positive correlation between DNA methylation and expression in some cell types [37, 38]. Dynamic DNA methylation in a gene body might lead to alternative splicing [39].

In addition, by combing genome-wide RNA expression data, we were also able to explore the response of gene expression to DNA methylation alterations induced by tobacco smoking. RNA-seq analysis indicated that RNA expression changes caused by smoking were associated mainly with immunosuppression and lymphocyte-related pathways, which is consistent with previous report [40]. It was proposed that tobacco smoking increases the number of alveolar macrophages (AMs) by several fold and induces cells to express more lysosomal enzymes and secrete elastase, which may damage the connective tissue and parenchymal cells of the lung, leading to chronic obstructive pulmonary disease (COPD) [5]. Ferson et al. pointed that natural killer (NK) cell activity against cultured melanoma and other cancer cells was significantly reduced in smokers compared with nonsmokers [4, 41]. In our study, the cytokine concentration comparison results showed that VEGF concentrations increased significantly in smokers compared with nonsmokers, which is consistent with a previous report [42]. In addition, nicotine can promote the secretion of VEGF in human trophoblast cells by reducing sFlt1 secretion, up-regulating VEGF transcription, and improving the proliferation and tube formation of HUVEC cells under hypoxic conditions [43]. Roybal et al. reported that cancer-associated fibroblasts (CAF) isolated from murine lung adenocarcinoma secreted rich amounts of VEGF to enhance tumor cell invasion [44]. All these findings suggest that smokers’ increased risk of lung disease and cancer may be attributable partly to the effects of cigarette smoke on the immune system.

The potential limitations of this study deserve some attentions. First, although we had a relatively large sample of male Chinese Han smokers for the WGBS and RNA-seq analysis, it was still of limited sample size. Our power analysis indicated that, with a sample size of 36 per group, we would have at least 85% power to detect a significant difference for both DNA methylation and RNA-seq analysis using a two-sided two-sample t test at the 5% level of significance. Second, the difference in DNAm distribution between cell types is greater than the difference between individuals [45]. We acknowledge that whole blood DNA represents a mixture of DNA from different cell populations and different cells may show different methylation patterns. However, methylation patterns still could be used as biomarkers for tobacco smoking exposure [27]. We did adjust the variation in cellular composition by using the algorithm developed by Houseman et al. [46] in our study. Further research will use methylation patterns of purified cells to identify subjects with clinically relevant reactions to smoke or other respiratory toxicants, thereby identifying patients at risk of development or in early stages of diseases. Third, genome methylation is also affected by external and internal factors. Although we could not eliminate these confounding factors from the analysis, we did try to minimize the effects of these potential confounding factors, such as recruiting subjects with almost same lifestyle and lived in the same area. Finally, it should be noted that, as for the cell line-derived maps, we could obtain cell-line-derived epigenetic markers and target genes of enhancers only from consortia such as NIH Roadmap Epigenomics Mapping Consortium. Although DNA epigenetic modification may also be controlled by genotype [47], this research focused primarily on DNA methylation changes without considering equally important changes at the genotype level. In future studies, we will further explore the effect of genotype on smoking-associated DNA methylation.

## Conclusions

In conclusion, we profiled the whole blood DNA methylome and whole-transcriptome sequencing in humans with the goal of identifying molecular changes potentially associated with tobacco smoking. By comparing smokers and nonsmokers, we found that smoking-induced DMRs have  different distribution patterns in the hypermethylation and hypomethylation regions. Further, our functional and correlation analyses of integrated epigenetic and transcriptomic data revealed that the completion of genome-wide maps in the field offered a refinement of our understanding of the molecular mechanisms underlying the response to tobacco smoking and its harmful effect.

## Materials and methods

### Description of samples

A large-scale study of the prevalence of cigarette smoking and nicotine dependence was conducted in Shanxi Province of China by our laboratory from June 2012 to January 2014 [48]. All subjects were recruited from local community hospitals when they visited those hospitals for their annual health examinations in the city of Jincheng. Within this city, coal mining is one of the major industries and many people engaged in the coal mining-related industry  were males and smoke. Furthermore, their living styles were relatively more uniform, which made this study population less variable and easy to control other potential variables. Participants were excluded if they had a clinical diagnosis of a mental disorder such as Alzheimer’s disease, major depression, or schizophrenia [49]. For each participant, we collected personal information such as age, sex, education, marital status, annual family income, smoking status, lifestyle features, and medical history. For details, please refer to our previous reports [48, 50, 51]. After providing a detailed explanation of the research project and process, written informed consent was obtained from each participant. The study and all questionnaires used in the study were approved by the Ethics Committee of the First Affiliated Hospital of Zhejiang University.

From these subjects, we selected 36 smokers and 36 nonsmokers for WGBS, and all participants were males. The criteria were as follows: (1) smokers were those who smoked at least 20 cigarettes per day. Nonsmokers were those who had smoked fewer than 100 cigarettes in their lifetimes [52, 53]; (2) there was no significant difference in age or Body Mass Index (BMI) between the smoker and nonsmoker groups; and (3) half of the subjects in each group were local miners, as Jincheng is a well-known coal mining area in China. In addition, we performed RNA-seq analysis on 38 smokers and 37 nonsmokers. Finally, we collected fresh plasma samples from 9 smokers and 13 healthy individuals for cytokine measurement from the sample of WGBS. Tables 1 and Additional file 1: Table S3 list the detailed demographic characteristics of the samples included in this study.

### Whole-genome bisulfite sequencing

Genomic DNA (gDNA) was prepared from whole blood samples using a Gentra Puregene Blood Kit (Qiagen) according to the manufacturer’s instructions and was stored at -80 °C until used. For library construction, we first mixed 200 ng of gDNA with 1 ng of unmethylated λ phage DNA and then used Ultrasound Generator Covaris S220 to shear the DNA into small fragments. After purification, adenosine was added to the 3′ ends of the fragmented DNA with a size of approximately 300 bp for end-repairing and connected with TruSeq adaptors (Illumina). Adapter-ligated DNA fragments were then treated with bisulfite using the EZ DNA methylation kit (Zymo Research) and PCR amplified. The PCR conditions were as follows: 45 s at 98 °C, then 10 cycles at 98 °C for 15 s, 65 °C for 30 s, and 72 °C for 30 s, and ending with 72 °C for 1 min. KAPA HiFi HotStart Uracil + DNA polymerase (Kapa Biosystems) was used to enrich the bisulfite-converted DNA through several PCR cycles. The quality of each library was quantified by Qubit 2.0 (Life Technology) and Agilent 2100 Bioanalyzer. DNA sequencing was conducted on the HiSeq X Ten platform using standard Illumina protocols.

### Data processing and differential DNA methylation analysis

Read mapping: For raw reads, Cutadapt (v. 1.18) [54] was used to delete adaptor sequences, and Trimmomatic (v. 0.33) [55] was employed to remove low-quality bases and adapter-less reads. Briefly, bases with a quality score of < 3 bp were pruned. Then, we used a 4-bp sliding window to scan the reads from the 5′ end to the 3′ end. Once the average quality score in the sliding window was < 15, all the reads from 5′ → 3′ were removed. After quality trimming, read sizes < 36 bp were excluded. Bismark (v. 0.16.1) [56] was used to map the generated pure sequencing reads with default parameter settings against the bisulfite-converted hg19 reference genome and remove all duplicate reads.

We first combined the CpG methylation information from DNA double strands. Because of the mixture of whole blood cells, we defined the methylation level as the number of methylated C reads divided by the total number of C reads at this site under the mCG sequence context [57]:

$$R_{{{\text{maverage}}}} = \frac{{{\text{Nmc}}}}{{{\text{Nmc}} + {\text{Nnmc}}}}{* }100{\% }$$

where Nmc is the number of methylated C reads, and Nnmc is the number of unmethylated C reads at this specific site.

In addition, because the extent of CpG methylation might be affected by the SNP overlapping with the CpG site of interest, we excluded those CpGs overlapped with the SNPs at a minor allele frequency (MAF) > 5%.

### DMR identification

In order to identify candidate DMRs, we first smoothed each CpG site by using bsseq (v 0.10) in the Bsmooth package [58], then calculated the significance for each of them and merged all adjacent significant CpG sites into DMRs. Each CpG site was smoothed in a window of minimum width of 1000 bp with at least 11 CpGs included; if a gap between two CpGs exceeded 2000 bp, smoothing was discontinued [21]. The relation between smoking and the extent of methylation of each CpG was examined using linear regression in R (v 3.4.0), with adjustment for potential confounders such as age, BMI, smoking status, working conditions, and blood cell composition. We used reference data in EpiDISH [59] to estimate the proportions of six blood cell subtypes (B cells, natural killer cells, CD4 + T cells, CD8 + T cells, monocytes, and granulocytes) in the whole blood samples. Next, the Comb-p software was used to discover candidate DMRs with a *P* value < 0.05 for all adjacent CpG sites included through the whole genome [60]. Each DMR to be included in the analysis had to meet the following two conditions: (1) it contained at least 5 CpG sites with each *P* value < 0.05 and (2) the distance between two adjacent CpG sites was ≤ 200 bp. We calculated the Stouffer–Liptak–Kechris (slk) corrected *P* value [61] for each DMR by Comb-p and then determined the hypermethylated and hypomethylated DMRs.

### Enrichment analysis and roadmap epigenomics annotation of DMRs

In order to determine whether smoking-associated DMRs were significantly enriched or depleted in specific regions of the genome, we adopted a random shuffling approach to calculate the significance of DMR enrichment in the regions of interest (ROI). All gene structures and CpG island annotations were downloaded from UCSC Hg19 Genome Browser tracks (https://www.genome.ucsc.edu/cgi-bin/hgTables). The ROIs were defined as follows: (1) gene body included 5′-UTR, exon, intron, and 3′-UTR. If the areas were not located in the above regions, they were defined as intergenic; (2) the relevant areas of CpG islands were defined as CpG islands (CGIs), CpG shores (2-kb regions adjacent upstream and downstream to CGIs), and CpG shelves (2-kb regions adjacent upstream and downstream of CpG shores or 4-kb regions adjacent upstream and downstream to CGIs). Any areas not belonging to the above regions were defined as open seas; (3) promoters were defined as the region of 1.5 kb upstream to 500 bp downstream of all gene transcription start sites (TSSs) based on the Human Genome Reference GRCH37. To define the epigenomic characteristics of smoking-DMRs (SM-DMRs), we downloaded the histone modification ChIP-seq peaks and chromatin state information of the reference 15-state epigenome model for 20 blood cells and two lung tissues from the NIH Roadmap Epigenomics Mapping Consortium (https://egg2.wustl.edu/roadmap/data/byFileType/chromhmmSegmentations/ChmmModels/coreMarks/jointModel/final/) [62] and used them for enrichment analysis.

For each DMR, 1000 matching regions were selected randomly from the whole genome (same CpG numbers and ± 5% difference region length as DMRs). Then, BEDTools was used to calculate the number of overlaps of these randomly generated matching background region sets with the ROIs [63]. An empirical null distribution was determined by repeating this process 1000 times. Finally, the empirical *P* value was calculated as (*r* + 1)/(*n* + 1), where n is the number of replicated samples that were simulated, and r is the number of these replicates that produces a test statistic greater than or equal to that calculated for the actual data [64]. The number of DMRs overlapped with target features divided by the average number of overlaps resulting from the randomizations 1000 times was defined as the fold change [21].

### Pathway enrichment analysis of DMR-related genes

To annotate the biological function of each DMR, we defined a DMR-related gene as a potential one if its upstream 2000 bp to downstream 1000 bp overlapped a known gene. We annotated the DMR genes (DMGs) and performed Kyoto Encyclopedia of Genes and Genomes (KEGG) by clusterProfiler [65]. The hypergeometric distribution was used to test the significance of functional categories in DMGs, and the pathway/Gene Ontology (GO) term satisfying a *P* value ≤ 0.05 was defined as significant in the pathway/GO term enriched by DMGs.

### RNA sequencing

To determine whether the differential methylation sites or regions in smokers vs. nonsmokers were related to potential biological effects on gene expression, we performed RNA-seq analysis on 75 males, among which 30 participants (12 smokers and 18 nonsmokers) already had WGBS data collected. Total RNA was extracted from the whole blood by RNeasy micro kit with Trizol. For the RNA-seq libraries, the RIN of each sample was required to be > 7. RNA quality was verified using Agilent 2100 Bioanalyzer (Agilent Technologies) and Nanodrop 2000. Sequencing was performed on Illumina HiSeqX (150 bp read pairs) using the standard parameters and 200 cycles of TruSeq SBS kit.

### RNA data processing and differential expression analysis

FastQC (v 0.11.9) was used for quality control and removal of adaptors (http://www.bioinformatics.bbsrc.ac.uk/projects/fastqc). We used HISAT2 (v 2.1.0) and StringTie (v 2.1.3) to align and map the paired-end reads to Human Genome Reference GRCH37 [66–68]. Transcripts expressed at ≥ 1 count per million reads in our samples were included for analysis. We used the Voom method in the limma package to normalize the raw counts (v 3.44.3) [69]. Differentially expressed genes (DEGs) in smokers and nonsmokers were detected with a linear regression model by including age, BMI, and working conditions as covariates.

### Correlation analysis between DMRs’ methylation and target gene expression

To determine whether the differential transcriptions in smokers and nonsmokers reflect smoking-induced epigenetic changes, we not only analyzed the gene where the DMR was located but also its distal regulated regions. In addition, we defined the DMR directly adjacent to a promoter (1.5-kb upstream to 500-bp downstream of TSS in Human Reference Genome GRCH37) as a **“**promoter-associated DMR.” To identify the enhancer and its target genes, we downloaded files from Genehancer database [70] and identified all significant DMRs that overlapped with these **“**enhancer associated DMR.” To better capture the subtle changes in gene expression related to SM-DMRs, all differentially expressed transcripts with *P* value < 0.05 and DMRs with *P* value ≤ 1.00 × 10^–4^ were included for next analysis. For the extent of methylation of each DMR, because it contains at least 5 CpG sites, we calculated the average methylation of all CpG sites within the region.

To determine the correlation between methylation and expression, we calculated DMR methylation and log2-transformed target gene expression as DMR-DEG pairs for each individual participant in 30 subjects with both WGBS and RNA-seq data (12 smokers and 18 nonsmokers). For each pair, the significance of the correlation was calculated by the Spearman correlation test, and the cutoff *P* value was set to 0.05. Smoking-related gene–disease correlations were analyzed by DisGeNET database in Metascape [71].

#### Multiple cytokine immunoassay

For cytokine measurement, we collected fresh plasma from 22 male WGBS subjects (see Additional file 1: Table S3) using the Bio-Plex Pro Human Cytokine 27-Plex Immunoassay kit. The Bio-Plex Manager software 6.0 (Bio-Rad) was used to quantify 27 blood cytokines on the Bio-Plex 200 Instrument. Finally, the log_2_-transformed cytokine fluorescence intensity was used to perform difference analysis between smokers and nonsmokers by linear regression in R with age, BMI, and working condition as covariates.

#### Statistical analysis

The free opensource statistical tools for the R software version 3.4 were used for all statistical analyses. Differences in age and BMI were analyzed using Wilcoxon rank sum test. A difference in working condition distribution was tested using Chi-squared test. The differential methylation (DM) detection procedure was implemented by linear regression, with adjustment for potential confounders such as age, BMI, working conditions, and blood cell compositions. Candidate DMRs contained at least 5 CpG sites (*P* value < 0.05) and with a distance ≤ 200 bp between two adjacent CpG sites. To better capture the subtle changes in gene expression related to smoking DMRs, all DMRs with a Stouffer–Liptak–Kechris (slk) corrected *P* value ≤ 1.00 × 10^–4^ and all differentially expressed transcripts with *P* value < 0.05 were included for all analyses reported here. For each DMR-DEG pair, the correlation was calculated by the Spearman correlation test. *P* < 0.05 was considered statistically significant.

## Supplementary Information

**Additional file 1.** **Table S1.** Quality control and alignment of WGBS. **Table S2.** Quality control and alignment of RNA-seq. **Table S3.** Sample characteristics of cytokine. **Table S4.** Genome-wide significant SM-DMRs (Top10). **Table S5.** KEGG pathway enrichment analysis of Hypermethylated DMRs-related gene (Top 15 pathways). **Table S6.** KEGG pathway enrichment analysis of Hypomethylated DMRs-related gene (Top 15 pathways). **Table S7.** Genome-wide significant DEGs (Top10). **Table S8.** Hyper- and Hypo-DMGs with FDR < 0.05. **Table S9.** DEGs with FDR < 0.05. **Table S10.** 148 DMR-DEG pairs. **Table S11.** KEGG pathway enrichment analysis of common genes in methylation and mRNA datasets. **Table S12.** Difference in blood cytokine between smokers and nonsmokers.

## Data Availability

The datasets used and/or analyzed in the current study are available from the corresponding author on reasonable request.

## Abbreviations

BMI: Body Mass Index
COPD: Chronic obstructive pulmonary disease
CGIs: CpG islands
CI: Confidence interval
CVD: Cardiovascular disease
DEG: Differentially expressed gene
DMG: DMR gene
DMR: Differentially methylated region
gDNA: Genomic DNA
GO: Gene Ontology
KEGG: Kyoto Encyclopedia of Genes and Genomes
MAF: Minor allele frequency
RNA-seq: RNA-sequencing
ROI: Regions of interest
SM-DMRs: Smoking-DMRs
TSSs: Transcription start sites
VEGF: Vascular endothelial growth factor
WGBS: Whole-genome bisulfite sequencing
