# Supplementary material for: Genome-wide methylation and expression analyses reveal the epigenetic landscape of immune-related diseases for tobacco smoking
Source: Clin Epigenetics. 2021 Dec 9;13:215. doi: 10.1186/s13148-021-01208-0 (PMC8662854; doi:10.1186/s13148-021-01208-0)
Supplement: Supplementary file 1 — Additional file 1. Table S1. Quality control and alignment of WGBS. Table S2. Quality control and alignment of RNA-seq. Table S3. Sample characteristics of cytokine. Table S4. Genome-wide significant SM-DMRs (Top10). Table S5. KEGG pathway enrichment analysis of Hypermethylated DMRs-related gene (Top 15 pathways). Table S6. KEGG pathway enrichment analysis of Hypomethylated DMRs-related gene (Top 15 pathways). Table S7. Genome-wide significant DEGs (Top10). Table S8. Hyper- and Hypo-DMGs with FDR < 0.05. Table S9. DEGs with FDR < 0.05. Table S10. 148 DMR-DEG pairs. Table S11. KEGG pathway enrichment analysis of common genes in methylation and mRNA datasets. Table S12. Difference in blood cytokine between smokers and nonsmokers. [file 13148_2021_1208_MOESM1_ESM.docx]

**Additional file1**

Table S1. Quality control and alignment of WGBS

| Sample | Raw reads  (M) | Clean reads  (M) | Mapping reads (M) | Mapping rate | Auto Chrosome  ≥ 2X CpG (M) | Auto Chrosome  Depth (X) | Auto Chrosome  Coverage (%) |
| --- | --- | --- | --- | --- | --- | --- | --- |
| Smokers | 672.66 ± 64.08 | 594.81 ± 76.66 | 477.20 ± 68.55 | 80.10 ± 2.20 | 25.24 ± 0.39 | 12.44 ± 1.82 | 87.95 ± 1.37 |
| Nonsmokers | 685.24 ± 53.09 | 614.00 ± 51.93 | 481.63 ± 42.69 | 78.51 ± 3.92 | 25.39 ± 0.27 | 12.62 ± 0.96 | 88.47 ± 0.93 |
| Total | 678.95 ± 58.77 | 604.40 ± 65.72 | 479.41 ± 56.74 | 79.31 ± 3.25 | 25.32 ± 0.34 | 12.53 ± 1.45 | 88.21 ± 1.19 |

Note: Data are presented as mean±standard deviation (SD) for continuous variables; WGBS, Whole genome bisulfite Sequencing.

Table S2. Quality control and alignment of RNA-seq

| Sample | Raw reads (M) | Clean reads (M) | Mapping rate (%) |
| --- | --- | --- | --- |
| Smokers | 86.14 ± 6.13 | 86.05 ± 6.13 | 83.84 ± 3.84 |
| Nonsmokers | 87.51 ± 6.47 | 87.41 ± 6.46 | 82.67 ± 3.26 |
| Total | 86.81 ± 6.30 | 86.72 ± 6.29 | 83.26 ± 3.59 |

Note: Data are presented as mean±standard deviation (SD) for continuous variables; RNA-seq, RNA-sequencing.

Table S3. Sample characteristics of cytokine

| Characteristics | Smokers | Nonsmokers | Total |
| --- | --- | --- | --- |
| Sample size | 9 | 13 | 22 |
| Average age (SD) | 41.22 (2.86) | 41.23 (2.31) | 41.23 (2.49) |
| BMI (SD) | 24.76 (3.04) | 26.46 (3.93) | 25.77 (3.62) |
| CPD (SD) | 21.33 (3.31) | 0 | — |
| FTND (SD) | 4.56 (2.07) | 0 | — |
| Miner (Non-miners) | 5 (4) | 4 (9) | 9 (13) |

Note: —, not applicable.

Table S4. Genome-wide significant SM-DMRs (Top10)

| Chr | Start | End | Raw P value | Adj P value | DMR location | Functional prediction |
| --- | --- | --- | --- | --- | --- | --- |
| chr2 | 233283801 | 233285198 | 9.51E-12 | 1.67E-07 | AC068134.5 | pseudogene |
| chr2 | 233283801 | 233285198 | 9.51E-12 | 1.67E-07 | AC068134.6 | lincRNA |
| chr10 | 71709189 | 71710198 | 1.13E-09 | 2.74E-05 | COL13A1 | Protein coding |
| chr8 | 128854648 | 128855399 | 4.12E-09 | 0.0001344 | PVT1 | Processed transcript |
| chr3 | 170135419 | 170135979 | 1.08E-08 | 0.0004724 | CLDN11 | Protein coding |
| chr3 | 170135419 | 170135979 | 1.08E-08 | 0.0004724 | RP11-469J4.3 | lincRNA |
| chr17 | 60669344 | 60669615 | 1.28E-08 | 0.001154 | TLK2 | Protein coding |
| chr10 | 135042060 | 135043045 | 2.20E-08 | 0.0005462 | UTF1 | Protein coding |
| chr3 | 16685525 | 16686181 | 2.56E-08 | 0.000955 | DAZL | Protein coding |
| chr14 | 23484224 | 23484455 | 2.70E-08 | 0.002856 | SNORA73 | snoRNA |

Note: SM, smoking associated.

Table S5. KEGG pathway enrichment analysis of Hypermethylated DMRs-related gene (Top 15 pathways)

| ID | Description | P value | Gene ID |
| --- | --- | --- | --- |
| hsa04360 | Axon guidance | 1.72E-05 | *PLCG2/DCC/PAK4/PLXNA1/ROBO2/EPHA6/ABLIM3/EPHB6/ABLIM1/CAMK2G/LRRC4C/CFL1/KRAS/EFNB2/PARD6G/MAPK1/EPHA4/ROBO1/SEMA5A/PLXNA4/SEMA3A/PTK2* |
| hsa04724 | Glutamatergic synapse | 1.60E-04 | *GRIK4/GNAO1/PLCB1/SHANK3/GRM7/SHANK2/GRM5/ITPR2/SLC38A1/PLCB2/HOMER2/ADCY9/DLGAP1/GRIK1/MAPK1* |
| hsa04915 | Estrogen signaling pathway | 4.18E-04 | *CREB3L1/GNAO1/PLCB1/NCOA1/ESR1/GPER1/CALML5/KRAS/ITPR2/AKT3/PLCB2/SHC4/ADCY9/MAPK1/FKBP5/GABBR2* |
| hsa04144 | Endocytosis | 5.55E-04 | *AP2A2/IQSEC3/ZFYVE9/SH3GL3/EPN2/AP2B1/HGS/NEDD4L/LDLR/RAB8A/AGAP1/SMAP1/AGAP3/MVB12B/IL2RA/GIT2/RAB35/SNX6/PARD6G/WIPF1/CYTH3/SMURF1/PSD3* |
| hsa04071 | Sphingolipid signaling pathway | 8.19E-04 | *ABCC1/PLCB1/PPP2R2A/SGMS1/CERS2/KRAS/AKT3/PPP2R3C/PPP2R5E/PLCB2/BID/MAPK1/PPP2CA/ASAH1* |
| hsa04152 | AMPK signaling pathway | 8.90E-04 | *PFKP/CREB3L1/STK11/RAB8A/PPP2R2A/AKT3/PPP2R3C/PPP2R5E/PFKFB4/SCD5/PPP2CA/PRKAG2/FBP2/FBP1* |
| hsa04730 | Long-term depression | 1.27E-03 | *PRKG1/GNAO1/PLCB1/GRID2/KRAS/ITPR2/PLCB2/MAPK1/PPP2CA* |
| hsa04012 | ErbB signaling pathway | 1.34E-03 | *PLCG2/PAK4/CRKL/CAMK2G/KRAS/AKT3/SHC4/NRG4/CRK/MAPK1/PTK2* |
| hsa04922 | Glucagon signaling pathway | 2.64E-03 | *PFKP/CREB3L1/PLCB1/CALML5/CAMK2G/ITPR2/AKT3/SLC2A1/PLCB2/PRKAG2/FBP2/FBP1* |
| hsa04928 | Parathyroid hormone synthesis, secretion and action | 2.64E-03 | *CREB3L1/PDE4A/PLCB1/RUNX2/RXRA/LRP6/ITPR2/VDR/PLCB2/ADCY9/PDE4C/MAPK1* |
| hsa04261 | Adrenergic signaling in cardiomyocytes | 2.65E-03 | *CREB3L1/PLCB1/PPP2R2A/CALML5/CAMK2G/AKT3/PPP2R3C/PPP2R5E/RPS6KA5/PLCB2/ADCY9/PIK3R5/CACNB4/MAPK1/PPP2CA* |
| hsa04720 | Long-term potentiation | 2.79E-03 | *PLCB1/CALML5/CAMK2G/GRM5/KRAS/ITPR2/PLCB2/MAPK1/RPS6KA2* |
| hsa05135 | Yersinia infection | 2.84E-03 | *DOCK1/ARHGEF7/MAP2K6/CRKL/ZAP70/GIT2/AKT3/WASF2/CRK/WIPF1/MAPK1/RPS6KA2/PTK2* |
| hsa01522 | Endocrine resistance | 4.19E-03 | *ESR1/E2F3/GPER1/KRAS/AKT3/JAG2/SHC4/ADCY9/MAPK1/DLL1/PTK2* |
| hsa04114 | Oocyte meiosis | 4.63E-03 | *PLK1/RBX1/CALML5/CAMK2G/ITPR2/PPP2R5E/CPEB1/ADCY9/MAPK1/PPP2CA/FBXW11/RPS6KA2/MAD1L1* |

Table S6. KEGG pathway enrichment analysis of Hypomethylated DMRs-related gene (Top 15 pathways)

| ID | Description | P value | Gene ID |
| --- | --- | --- | --- |
| hsa05202 | Transcriptional misregulation in cancer | 9.66E-04 | *JMJD1C/FLT3/FLT1/BCL11B/JUP/IL2RB/ZBTB16/MDM2/IL1R2/MYCN/MEIS1* |
| hsa04725 | Cholinergic synapse | 1.47E-03 | *ADCY4/ADCY9/CACNA1A/KCNQ2/KCNQ3/GNA11/PLCB1/KCNQ5* |
| hsa05032 | Morphine addiction | 1.89E-03 | *ADCY4/ADCY9/CACNA1A/GABRA2/GABBR2/PDE3B/GABRA1* |
| hsa04015 | Rap1 signaling pathway | 2.55E-03 | *RAP1GAP/FLT1/ADCY4/ADCY9/ACTG1/PARD6G/INSR/EFNA5/EVL/F2RL3/PLCB1* |
| hsa04923 | Regulation of lipolysis in adipocytes | 4.14E-03 | *ADCY4/ADCY9/INSR/NPY1R/PDE3B* |
| hsa04022 | cGMP-PKG signaling pathway | 5.16E-03 | *KCNMA1/ADCY4/ADCY9/INSR/MYLK4/PDE3B/GNA11/PLCB1/GATA4* |
| hsa04742 | Taste transduction | 5.40E-03 | *ADCY4/CACNA1A/GABRA2/GRM4/GABBR2/GABRA1* |
| hsa04724 | Glutamatergic synapse | 6.65E-03 | *SHANK2/ADCY4/ADCY9/CACNA1A/GRM4/PLA2G4A/PLCB1* |
| hsa04727 | GABAergic synapse | 7.56E-03 | *ADCY4/ADCY9/CACNA1A/GABRA2/GABBR2/GABRA1* |
| hsa04919 | Thyroid hormone signaling pathway | 8.35E-03 | *MED24/ACTG1/NCOA2/MED27/MDM2/PLCB1/GATA4* |
| hsa04927 | Cortisol synthesis and secretion | 8.42E-03 | *ADCY4/ADCY9/CACNA1G/GNA11/PLCB1* |
| hsa03060 | Protein export | 9.60E-03 | *IMMP1L/IMMP2L/HSPA5* |
| hsa04611 | Platelet activation | 1.04E-02 | *ADCY4/ADCY9/ACTG1/MYLK4/PLA2G4A/F2RL3/PLCB1* |
| hsa04925 | Aldosterone synthesis and secretion | 1.19E-02 | *ADCY4/ADCY9/DAGLA/CACNA1G/GNA11/PLCB1* |
| hsa04020 | Calcium signaling pathway | 1.28E-02 | *ADCY4/ADCY9/CACNA1A/ERBB4/MYLK4/TPCN1/CACNA1G/GNA11/PLCB1* |

Table S7. Genome-wide significant DEGs (Top10)

| Gene | Log_2_(FC) | Ave Expr  (Smokers-Nonsmokers) | P value | Adj P value |
| --- | --- | --- | --- | --- |
| *GPR15* | 2.25 | 1.83 | 2.54E-14 | 4.73E-10 |
| *LRRN3* | 1.10 | 2.56 | 7.92E-08 | 7.39E-04 |
| *IL2RA* | 0.67 | 1.17 | 3.80E-07 | 2.36E-03 |
| *CLDND1* | 0.27 | 4.83 | 1.68E-06 | 6.41E-03 |
| *P2RY6* | 1.02 | -0.35 | 1.72E-06 | 6.41E-03 |
| *AC104809.3* | -0.99 | -1.01 | 5.51E-06 | 1.56 E-02 |
| *CYP4F22* | -0.75 | 0.14 | 6.73E-06 | 1.56 E-02 |
| *COLGALT2* | -0.73 | 2.07 | 7.35E-06 | 1.56E-02 |
| *CHAF1B* | -0.46 | 0.77 | 8.10E-06 | 1.56E-02 |
| *THEM4* | 0.40 | 3.42 | 9.39E-06 | 1.56E-02 |

Table S8. Hyper- and Hypo-DMGs with FDR < 0.05

| Chr | Start | End | P value | Adj P value | Gene ID | Gene name | Type | Direction |
| --- | --- | --- | --- | --- | --- | --- | --- | --- |
| chr2 | 233283801 | 233285198 | 9.51E-12 | 1.67E-07 | ENSG00000204121 | *AC068134.5* | pseudogene | Hypo |
| chr2 | 233283801 | 233285198 | 9.51E-12 | 1.67E-07 | ENSG00000237087 | *AC068134.6* | lincRNA | Hypo |
| chr10 | 71709189 | 71710198 | 1.13E-09 | 2.74E-05 | ENSG00000197467 | *COL13A1* | protein_coding | Hyper |
| chr8 | 128854648 | 128855399 | 4.12E-09 | 1.34E-04 | ENSG00000249859 | *PVT1* | processed_transcript | Hyper |
| chr3 | 170135419 | 170135979 | 1.08E-08 | 4.72E-04 | ENSG00000013297 | *CLDN11* | protein_coding | Hyper |
| chr3 | 170135419 | 170135979 | 1.08E-08 | 4.72E-04 | ENSG00000242578 | *RP11-469J4.3* | lincRNA | Hyper |
| chr10 | 135042060 | 135043045 | 2.20E-08 | 5.46E-04 | ENSG00000171794 | *UTF1* | protein_coding | Hyper |
| chr3 | 16685525 | 16686181 | 2.56E-08 | 9.55E-04 | ENSG00000092345 | *DAZL* | protein_coding | Hyper |
| chr17 | 60669344 | 60669615 | 1.28E-08 | 1.15E-03 | ENSG00000146872 | *TLK2* | protein_coding | Hyper |
| chr21 | 43932446 | 43932979 | 4.02E-08 | 1.84E-03 | ENSG00000160190 | *SLC37A1* | protein_coding | Hyper |
| chr13 | 100549860 | 100550189 | 3.01E-08 | 2.24E-03 | ENSG00000125246 | *CLYBL* | protein_coding | Hyper |
| chr1 | 20985181 | 20985505 | 4.92E-08 | 3.71E-03 | ENSG00000244038 | *DDOST* | protein_coding | Hypo |
| chr5 | 161989636 | 161989918 | 4.39E-08 | 3.80E-03 | ENSG00000254186 | *RP11-167P20.1* | lincRNA | Hyper |
| chr8 | 1527988 | 1528616 | 1.14E-07 | 4.43E-03 | ENSG00000198010 | *DLGAP2* | protein_coding | Hyper |
| chr8 | 1527988 | 1528616 | 1.14E-07 | 4.43E-03 | ENSG00000253267 | *RP11-666I19.2* | antisense | Hyper |
| chr12 | 124217937 | 124218331 | 8.19E-08 | 5.08E-03 | ENSG00000185344 | *ATP6V0A2* | protein_coding | Hyper |
| chr22 | 42594697 | 42594962 | 5.78E-08 | 5.33E-03 | ENSG00000100207 | *TCF20* | protein_coding | Hyper |
| chr11 | 18210560 | 18211323 | 1.67E-07 | 5.34E-03 | ENSG00000255138 | *GLTPP1* | pseudogene | Hyper |
| chr11 | 18210560 | 18211323 | 1.67E-07 | 5.34E-03 | ENSG00000224002 | *AC090099.2* | pseudogene | Hyper |
| chr11 | 18210560 | 18211323 | 1.67E-07 | 5.34E-03 | ENSG00000255470 | *RP11-113D6.6* | protein_coding | Hyper |
| chr19 | 33288425 | 33288655 | 7.02E-08 | 7.45E-03 | ENSG00000173809 | *TDRD12* | protein_coding | Hyper |
| chr16 | 83986701 | 83987734 | 3.37E-07 | 7.96E-03 | ENSG00000140961 | *OSGIN1* | protein_coding | Hyper |
| chr1 | 112814747 | 112815162 | 1.37E-07 | 8.04E-03 | ENSG00000231246 | *RP5-965F6.2* | lincRNA | Hyper |
| chr1 | 78258975 | 78259532 | 1.85E-07 | 8.10E-03 | ENSG00000180488 | *FAM73A* | protein_coding | Hyper |
| chr20 | 8659135 | 8659584 | 1.57E-07 | 8.53E-03 | ENSG00000182621 | *PLCB1* | protein_coding | Hypo |
| chr3 | 98239609 | 98240661 | 4.02E-07 | 9.30E-03 | ENSG00000080822 | *CLDND1* | protein_coding | Hypo |
| chr3 | 98239609 | 98240661 | 4.02E-07 | 9.30E-03 | ENSG00000080819 | *CPOX* | protein_coding | Hypo |
| chr3 | 98239609 | 98240661 | 4.02E-07 | 9.30E-03 | ENSG00000248839 | *RP11-227H4.5* | antisense | Hypo |
| chr14 | 32267035 | 32267309 | 1.09E-07 | 9.66E-03 | ENSG00000151413 | *NUBPL* | protein_coding | Hyper |
| chr3 | 173155301 | 173155608 | 1.22E-07 | 9.71E-03 | ENSG00000169760 | *NLGN1* | protein_coding | Hyper |
| chr9 | 139003115 | 139003560 | 1.78E-07 | 9.73E-03 | ENSG00000260193 | *RP11-83N9.5* | lincRNA | Hypo |
| chr15 | 22969652 | 22970082 | 1.90E-07 | 1.08E-02 | ENSG00000068793 | *CYFIP1* | protein_coding | Hyper |
| chr4 | 26258941 | 26259143 | 9.11E-08 | 1.10E-02 | ENSG00000168214 | *RBPJ* | protein_coding | Hyper |
| chr7 | 139055167 | 139055802 | 3.16E-07 | 1.21E-02 | ENSG00000146963 | *C7orf55-LUC7L2* | protein_coding | Hyper |
| chr7 | 139055167 | 139055802 | 3.16E-07 | 1.21E-02 | ENSG00000269955 | *LUC7L2* | protein_coding | Hyper |
| chr5 | 156036413 | 156036883 | 2.35E-07 | 1.22E-02 | ENSG00000170624 | *SGCD* | protein_coding | Hyper |
| chr10 | 134143859 | 134145216 | 7.27E-07 | 1.30E-02 | ENSG00000148814 | *LRRC27* | protein_coding | Hyper |
| chr10 | 134143859 | 134145216 | 7.27E-07 | 1.30E-02 | ENSG00000165752 | *STK32C* | protein_coding | Hyper |
| chr6 | 134964557 | 134965043 | 2.92E-07 | 1.46E-02 | ENSG00000227723 | *CTA-31J9.2* | lincRNA | Hyper |
| chr6 | 134964557 | 134965043 | 2.92E-07 | 1.46E-02 | ENSG00000232310 | *RP11-557H15.4* | lincRNA | Hyper |
| chr5 | 9380421 | 9380961 | 3.26E-07 | 1.47E-02 | ENSG00000248537 | *CTD-2201E9.4* | antisense | Hyper |
| chr5 | 9380421 | 9380961 | 3.26E-07 | 1.47E-02 | ENSG00000112902 | *SEMA5A* | protein_coding | Hyper |
| chr5 | 372991 | 373651 | 4.19E-07 | 1.54E-02 | ENSG00000063438 | *AHRR* | protein_coding | Hypo |
| chr11 | 2333822 | 2334169 | 2.36E-07 | 1.65E-02 | ENSG00000064201 | *TSPAN32* | protein_coding | Hyper |
| chr20 | 58308525 | 58309167 | 4.53E-07 | 1.71E-02 | ENSG00000087495 | *PHACTR3* | protein_coding | Hyper |
| chr14 | 63955059 | 63955459 | 2.88E-07 | 1.75E-02 | ENSG00000154001 | *PPP2R5E* | protein_coding | Hyper |
| chr16 | 88330761 | 88331201 | 3.29E-07 | 1.81E-02 | ENSG00000261273 | *LA16c-444G7.1* | lincRNA | Hyper |
| chr1 | 205375620 | 205375934 | 2.39E-07 | 1.84E-02 | ENSG00000186007 | *LEMD1* | protein_coding | Hypo |
| chr13 | 111867351 | 111868041 | 5.30E-07 | 1.86E-02 | ENSG00000102606 | *ARHGEF7* | protein_coding | Hyper |
| chr2 | 98962749 | 98963585 | 6.48E-07 | 1.88E-02 | ENSG00000227987 | *AC092675.4* | sense_intronic | Hyper |
| chr2 | 98962749 | 98963585 | 6.48E-07 | 1.88E-02 | ENSG00000144191 | *CNGA3* | protein_coding | Hyper |
| chr2 | 98962749 | 98963585 | 6.48E-07 | 1.88E-02 | ENSG00000222000 | *AC092675.3* | protein_coding | Hyper |
| chr3 | 182970727 | 182971058 | 2.57E-07 | 1.88E-02 | ENSG00000053524 | *MCF2L2* | protein_coding | Hypo |
| chr3 | 182970727 | 182971058 | 2.57E-07 | 1.88E-02 | ENSG00000176597 | *B3GNT5* | protein_coding | Hypo |
| chr12 | 48289327 | 48289770 | 3.62E-07 | 1.98E-02 | ENSG00000205537 | *RP11-89H19.1* | antisense | Hyper |
| chr12 | 48289327 | 48289770 | 3.62E-07 | 1.98E-02 | ENSG00000111424 | *VDR* | protein_coding | Hyper |
| chr2 | 238471843 | 238472289 | 3.76E-07 | 2.04E-02 | ENSG00000222449 | *RNU6-1140P* | snRNA | Hypo |
| chr12 | 31954468 | 31955055 | 5.24E-07 | 2.16E-02 | ENSG00000252421 | *RNU6-1069P* | snRNA | Hypo |
| chr14 | 58795356 | 58795667 | 2.83E-07 | 2.20E-02 | ENSG00000032219 | *ARID4A* | protein_coding | Hyper |
| chr2 | 25286742 | 25287128 | 3.54E-07 | 2.22E-02 | ENSG00000084710 | *EFR3B* | protein_coding | Hypo |
| chr11 | 84630475 | 84630926 | 4.53E-07 | 2.43E-02 | ENSG00000150672 | *DLG2* | protein_coding | Hyper |
| chr3 | 195955585 | 195956341 | 8.41E-07 | 2.69E-02 | ENSG00000163959 | *SLC51A* | protein_coding | Hyper |
| chr3 | 195955585 | 195956341 | 8.41E-07 | 2.69E-02 | ENSG00000161217 | *PCYT1A* | protein_coding | Hyper |
| chr10 | 94448693 | 94449041 | 3.94E-07 | 2.73E-02 | ENSG00000152804 | *HHEX* | protein_coding | Hyper |
| chr2 | 237362471 | 237362883 | 4.75E-07 | 2.78E-02 | ENSG00000132321 | *IQCA1* | protein_coding | Hyper |
| chr14 | 105619745 | 105620094 | 4.23E-07 | 2.92E-02 | ENSG00000184916 | *JAG2* | protein_coding | Hyper |
| chr14 | 105619745 | 105620094 | 4.23E-07 | 2.92E-02 | ENSG00000257622 | *RP11-44N21.4* | processed_transcript | Hyper |
| chr13 | 21606905 | 21607439 | 6.61E-07 | 2.99E-02 | ENSG00000150457 | *LATS2* | protein_coding | Hypo |
| chr17 | 63577904 | 63578666 | 1.04E-06 | 3.29E-02 | ENSG00000266076 | *CTD-2535L24.2* | protein_coding | Hyper |
| chr13 | 62602377 | 62602712 | 4.60E-07 | 3.31E-02 | ENSG00000229578 | *LINC00358* | lincRNA | Hyper |
| chr18 | 77080200 | 77080550 | 4.85E-07 | 3.34E-02 | ENSG00000166377 | *ATP9B* | protein_coding | Hyper |
| chr18 | 3179372 | 3179957 | 8.43E-07 | 3.47E-02 | ENSG00000101605 | *MYOM1* | protein_coding | Hypo |
| chr19 | 7162620 | 7162787 | 2.42E-07 | 3.49E-02 | ENSG00000171105 | *INSR* | protein_coding | Hypo |
| chr1 | 217100167 | 217100559 | 5.97E-07 | 3.66E-02 | ENSG00000196482 | *ESRRG* | protein_coding | Hyper |
| chr17 | 25756865 | 25757417 | 8.65E-07 | 3.76E-02 | ENSG00000266433 | *TBC1D3P5* | pseudogene | Hyper |
| chr7 | 3445482 | 3445923 | 6.94E-07 | 3.78E-02 | ENSG00000146555 | *SDK1* | protein_coding | Hyper |
| chr9 | 97385326 | 97385556 | 3.82E-07 | 3.99E-02 | ENSG00000165140 | *FBP1* | protein_coding | Hyper |
| chr22 | 32756257 | 32756703 | 7.47E-07 | 4.02E-02 | ENSG00000128276 | *RFPL3* | protein_coding | Hyper |
| chr22 | 32756257 | 32756703 | 7.47E-07 | 4.02E-02 | ENSG00000205853 | *RFPL3S* | protein_coding | Hyper |
| chr15 | 52553140 | 52553612 | 7.95E-07 | 4.04E-02 | ENSG00000128833 | *MYO5C* | protein_coding | Hyper |
| chr20 | 60571506 | 60571832 | 5.60E-07 | 4.12E-02 | ENSG00000130699 | *TAF4* | protein_coding | Hyper |
| chr22 | 21298911 | 21299426 | 9.13E-07 | 4.25E-02 | ENSG00000099942 | *CRKL* | protein_coding | Hyper |
| chr18 | 63438596 | 63439153 | 1.02E-06 | 4.38E-02 | ENSG00000081138 | *CDH7* | protein_coding | Hypo |
| chr19 | 16240664 | 16241168 | 9.33E-07 | 4.43E-02 | ENSG00000167461 | *RAB8A* | protein_coding | Hyper |
| chr19 | 16240664 | 16241168 | 9.33E-07 | 4.43E-02 | ENSG00000269243 | *CTD-2231E14.8* | antisense | Hyper |
| chr16 | 79812538 | 79812975 | 8.23E-07 | 4.50E-02 | ENSG00000260876 | *RP11-345M22.1* | lincRNA | Hyper |
| chr6 | 169073614 | 169073972 | 7.17E-07 | 4.78E-02 | ENSG00000112562 | *SMOC2* | protein_coding | Hyper |
| chr2 | 178963758 | 178963981 | 4.64E-07 | 4.96E-02 | ENSG00000249909 | *CYCTP* | pseudogene | Hyper |
| chr2 | 178963758 | 178963981 | 4.64E-07 | 4.96E-02 | ENSG00000128655 | *PDE11A* | protein_coding | Hyper |
| chr7 | 65576199 | 65576488 | 6.02E-07 | 4.97E-02 | ENSG00000249319 | *AC068533.7* | protein_coding | Hyper |

Table S9. DEGs with FDR < 0.05

| Gene ID | Gene name | logFC | P value | Adj P value | Direction | Type |
| --- | --- | --- | --- | --- | --- | --- |
| ENSG00000154165 | *GPR15* | 2.25 | 2.54E-14 | 4.73E-10 | up | protein_coding |
| ENSG00000173114 | *LRRN3* | 1.10 | 7.92E-08 | 7.39E-04 | up | protein_coding |
| ENSG00000134460 | *IL2RA* | 0.67 | 3.80E-07 | 2.36E-03 | up | protein_coding |
| ENSG00000080822 | *CLDND1* | 0.27 | 1.68E-06 | 6.41E-03 | up | protein_coding |
| ENSG00000171631 | *P2RY6* | 1.02 | 1.72E-06 | 6.41E-03 | up | protein_coding |
| ENSG00000226321 | *AC104809.3* | -0.99 | 5.51E-06 | 1.56E-02 | down | protein_coding |
| ENSG00000171954 | *CYP4F22* | -0.75 | 6.73E-06 | 1.56E-02 | down | protein_coding |
| ENSG00000198756 | *COLGALT2* | -0.73 | 7.35E-06 | 1.56E-02 | down | protein_coding |
| ENSG00000159259 | *CHAF1B* | -0.46 | 8.10E-06 | 1.56E-02 | down | protein_coding |
| ENSG00000159445 | *THEM4* | 0.40 | 9.39E-06 | 1.56E-02 | up | protein_coding |
| ENSG00000075234 | *TTC38* | -0.43 | 9.54E-06 | 1.56E-02 | down | protein_coding |
| ENSG00000004468 | *CD38* | -0.48 | 1.02E-05 | 1.56E-02 | down | protein_coding |
| ENSG00000138166 | *DUSP5* | -0.46 | 1.14E-05 | 1.56E-02 | down | protein_coding |
| ENSG00000180155 | *LYNX1* | -0.87 | 1.21E-05 | 1.56E-02 | down | protein_coding |
| ENSG00000134864 | *GGACT* | -0.58 | 1.25E-05 | 1.56E-02 | down | protein_coding |
| ENSG00000224356 | *RP11-151A6.4* | -0.67 | 1.61E-05 | 1.87E-02 | down | sense_intronic |
| ENSG00000077063 | *CTTNBP2* | 1.31 | 1.95E-05 | 2.14E-02 | up | protein_coding |
| ENSG00000136153 | *LMO7* | 0.31 | 2.15E-05 | 2.22E-02 | up | protein_coding |
| ENSG00000152495 | *CAMK4* | 0.36 | 2.35E-05 | 2.31E-02 | up | protein_coding |
| ENSG00000178562 | *CD28* | 0.35 | 2.66E-05 | 2.48E-02 | up | protein_coding |
| ENSG00000167680 | *SEMA6B* | 1.24 | 4.54E-05 | 4.03E-02 | up | protein_coding |
| ENSG00000158813 | *EDA* | 0.64 | 4.76E-05 | 4.04E-02 | up | protein_coding |
| ENSG00000163606 | *CD200R1* | 0.38 | 5.68E-05 | 4.40E-02 | up | protein_coding |
| ENSG00000138795 | *LEF1* | 0.49 | 5.84E-05 | 4.40E-02 | up | protein_coding |
| ENSG00000174348 | *PODN* | -0.80 | 5.89E-05 | 4.40E-02 | down | protein_coding |
| ENSG00000147044 | *CASK* | 0.24 | 6.35E-05 | 4.55E-02 | up | protein_coding |

Table S10. 148 DMR-DEG pairs

| DMR | Predicted target Gene | Direction | DMR P value | DEG | DEG P value |
| --- | --- | --- | --- | --- | --- |
| chr5:138471976-138472191 | *SIL1* | Hyper | 4.33E-05 | down | 1.60E-04 |
| chr8:145666690-145666802 | *TONSL* | Hyper | 3.26E-05 | down | 8.05E-04 |
| chr16:588842-589059 | *CAPN15* | Hyper | 3.72E-06 | down | 3.42E-03 |
| chr7:140414385-140414633 | *NDUFB2* | Hyper | 4.28E-05 | down | 3.45E-03 |
| chr17:415069-415609 | *VPS53* | Hyper | 1.80E-06 | down | 3.76E-03 |
| chr12:48289327-48289770 | *DDX23* | Hyper | 6.24E-05 | down | 4.08E-03 |
| chr12:108733790-108734067 | *CMKLR1* | Hyper | 7.81E-05 | down | 5.33E-03 |
| chr2:152746588-152746978 | *CACNB4* | Hyper | 1.76E-05 | down | 5.71E-03 |
| chr1:6327901-6328131 | *ACOT7* | Hyper | 5.08E-05 | down | 5.77E-03 |
| chr17:81043739-81044429 | *METRNL* | Hyper | 4.0E-05 | down | 5.89E-03 |
| chr10:95201042-95201479 | *MYOF* | Hyper | 2.56E-05 | down | 6.55E-03 |
| chr16:89959962-89960328 | *TCF25* | Hyper | 2.38E-05 | down | 7.03E-03 |
| chr19:10614564-10615030 | *KEAP1* | Hyper | 4.00E-05 | down | 7.73E-03 |
| chr3:160764268-160764590 | *PPM1L* | Hyper | 3.87E-05 | down | 8.75E-03 |
| chr20:62292231-62292644 | *RTEL1* | Hyper | 6.23E-05 | down | 8.76E-03 |
| chr18:29607978-29608129 | *RP11-53I6.2* | Hyper | 6.32E-05 | down | 9.13E-03 |
| chr19:1880091-1880444 | *ABHD17A* | Hyper | 1.70E-05 | down | 1.03E-02 |
| chr22:26984699-26985033 | *TPST2* | Hyper | 5.04E-06 | down | 1.04E-03 |
| chr1:92291397-92291719  chr1:92270210-92270406 | *TGFBR3* | Hyper  Hypo | 1.62E-05  8.64E-05 | down | 1.13E-02 |
| chr2:236942159-236942376 | *AGAP1* | Hyper | 1.13E-05 | down | 1.17E-02 |
| chr10:135037434-135038012 | *KNDC1* | Hyper | 9.44E-06 | down | 1.26E-02 |
| chr19:10572259-10572563 | *PDE4A* | Hyper | 4.01E-05 | down | 1.33E-02 |
| chr22:18602349-18602669 | *PEX26* | Hyper | 2.83E-05 | down | 1.40E-02 |
| chr4:6041075-6041492 | *JAKMIP1* | Hyper | 2.72E-05 | down | 1.40E-02 |
| chr9:139906078-139906343 | *ABCA2* | Hyper | 7.87E-05 | down | 1.41E-02 |
| chr4:83640103-83640191 | *SCD5* | Hyper | 2.98E-05 | down | 1.45E-02 |
| chr10:126413379-126413691 | *FAM53B* | Hyper | 9.67E-06 | down | 1.47E-02 |
| chr11:31994804-31995240 | *RCN1* | Hyper | 9.11E-06 | down | 1.53E-02 |
| chr14:24002493-24002806 | *RP11-66N24.4* | Hyper | 2.06E-05 | down | 1.63E-02 |
| chr8:67697643-67697930 | *C8orf44-SGK3* | Hyper | 4.08E-05 | down | 1.76E-02 |
| chr1:161513847-161514197 | *FCGR3A* | Hyper | 6.10E-05 | down | 1.76E-02 |
| chr22:26889389-26889577 | *TFIP11* | Hyper | 4.75E-05 | down | 1.89E-02 |
| chr19:11211598-11211889 | *LDLR* | Hyper | 6.43E-05 | down | 2.02E-02 |
| chr19:30158120-30158513 | *PLEKHF1* | Hyper | 6.91E-06 | down | 2.51E-02 |
| chr15:78277976-78278338 | *TBC1D2B* | Hyper | 4.77E-05 | down | 2.60E-02 |
| chr22:47040659-47040953 | *GRAMD4* | Hyper | 4.21E-05 | down | 2.64E-02 |
| chr12:113798078-113798201 | *PLBD2* | Hyper | 8.76E-05 | down | 2.81E-02 |
| chr1:161569127-161569601 | *FCGR2C* | Hyper | 6.10E-05 | down | 2.96E-02 |
| chr9:130646115-130646569 | *ST6GALNAC6* | Hyper | 1.44E-05 | down | 3.00E-02 |
| chr17:79247878-79248510 | *SLC38A10* | Hyper | 8.74E-05 | down | 3.09E-02 |
| chr16:57014552-57014951 | *CETP* | Hyper | 5.85E-05 | down | 3.09E-02 |
| chr2:98338140-98338371 | *ZAP70* | Hyper | 9.16E-05 | down | 3.09E-02 |
| chr12:120546982-120547244  chr12:120534669-120535068 | *RAB35* | Hyper  Hypo | 9.38E-06  7.10E-05 | down | 3.32E-02 |
| chr19:4045342-4045412 | *ZBTB7A* | Hyper | 6.88E-05 | down | 3.70E-02 |
| chr17:9569619-9569895 | *USP43* | Hyper | 5.39E-05 | down | 3.70E-02 |
| chr13:98117447-98117710 | *RAP2A* | Hyper | 3.20E-05 | down | 3.72E-02 |
| chr11:67024748-67025170 | *RAD9A* | Hyper | 8.47E-05 | down | 3.98E-02 |
| chr2:74375246-74375595 | *BOLA3* | Hyper | 9.63E-05 | down | 3.99E-02 |
| chr17:18173858-18174216 | *TOP3A* | Hyper | 2.21E-06 | down | 4.14E-02 |
| chr17:42335362-42335652 | *SLC4A1* | Hyper | 3.76E-06 | down | 4.15E-02 |
| chr7:150817808-150818362 | *AGAP3* | Hyper | 1.14E-05 | down | 4.21E-02 |
| chr3:49710490-49711033 | *APEH* | Hyper | 6.41E-05 | down | 4.45E-02 |
| chr4:893194-893669 | *GAK* | Hyper | 4.67E-05 | down | 4.68E-02 |
| chr3:121229839-121230152 | *POLQ* | Hyper | 8.25E-05 | down | 4.81E-02 |
| chr22:21349778-21350083 | *LZTR1* | Hyper | 8.26E-05 | down | 4.93E-02 |
| chr10:71709189-71710198 | *COL13A1* | Hyper | 1.13E-09 | down | 4.93E-02 |
| chr3:129290004-129290503 | *PLXND1* | Hyper | 4.92E-05 | down | 4.99E-02 |
| chr10:6055819-6056041 | *IL2RA* | Hyper | 4.63E-06 | up | 3.80E-07 |
| chr3:13898755-13899135 | *WNT7A* | Hyper | 5.74E-05 | up | 2.16E-04 |
| chr4:149312755-149313439 | *NR3C2* | Hyper | 3.80E-05 | up | 3.81E-04 |
| chr2:222312663-222313620 | *EPHA4* | Hyper | 7.02E-06 | up | 6.53E-04 |
| chr11:44839868-44840277 | *TSPAN18* | Hyper | 3.54E-06 | up | 8.06E-04 |
| chr17:6980364-6980673 | *CLEC10A* | Hyper | 4.56E-05 | up | 1.14E-03 |
| chr11:133827605-133827961 | *IGSF9B* | Hyper | 4.54E-05 | up | 1.53E-03 |
| chr8:128854648-128855399 | *PVT1* | Hyper | 4.12E-09 | up | 3.46E-03 |
| chr6:148613495-148613952 | *SASH1* | Hyper | 3.67E-05 | up | 3.77E-03 |
| chr6:111421622-111421694 | *SLC16A10* | Hyper | 2.30E-06 | up | 4.40E-03 |
| chr5:158285125-158285317 | *EBF1* | Hyper | 1.68E-05 | up | 4.55E-03 |
| chr1:52785827-52786037 | *ZFYVE9* | Hyper | 6.16E-05 | up | 4.83E-03 |
| chr10:61830401-61830708 | *ANK3* | Hyper | 3.53E-06 | up | 5.35E-03 |
| chr2:229913383-229913876 | *PID1* | Hyper | 2.84E-05 | up | 6.09E-03 |
| chr5:80417333-80417694 | *RASGRF2* | Hyper | 8.02E-05 | up | 7.24E-03 |
| chr7:90364297-90364719 | *CDK14* | Hyper | 2.92E-05 | up | 7.27E-03 |
| chr19:3043935-3044321 | *TLE2* | Hyper | 6.41E-05 | up | 7.35E-03 |
| chr10:116211360-116211478 | *ABLIM1* | Hyper | 5.93E-05 | up | 7.60E-03 |
| chr2:228155033-228155194 | *COL4A3* | Hyper | 1.15E-06 | up | 9.53E-03 |
| chr8:125379442-125379668 | *TMEM65* | Hyper | 6.56E-05 | up | 9.84E-03 |
| chr3:31798283-31798935 | *OSBPL10* | Hyper | 2.60E-05 | up | 1.19E-02 |
| chr12:111634863-111635345 | *CUX2* | Hyper | 5.24E-06 | up | 1.32E-02 |
| chr2:112899009-112899266 | *FBLN7* | Hyper | 6.32E-05 | up | 1.41E-02 |
| chr4:75150606-75151022 | *MTHFD2L* | Hyper | 2.82E-05 | up | 1.84E-02 |
| chr4:110608692-110609168 | *CASP6* | Hyper | 3.85E-05 | up | 2.23E-02 |
| chr14:69052050-69052169 | *RAD51B* | Hyper | 1.97E-05 | up | 2.24E-02 |
| chr1:196704950-196705234 | *CFH* | Hyper | 4.62E-06 | up | 2.57E-02 |
| chr7:145915873-145916241 | *CNTNAP2* | Hyper | 2.38E-06 | up | 2.94E-02 |
| chr17:67458221-67458561 | *MAP2K6* | Hyper | 2.64E-05 | up | 3.38E-02 |
| chr1:243968004-243968103 | *AKT3* | Hyper | 7.37E-05 | up | 4.09E-02 |
| chr7:1130696-1131155 | *GPER1* | Hyper | 3.77E-06 | up | 4.43E-02 |
| chr9:19375082-19375220 | *RP11-513M16.8* | Hyper | 2.25E-05 | up | 4.55E-02 |
| chr5:156381183-156381504 | *TIMD4* | Hyper | 2.64E-05 | up | 4.57E-02 |
| chr3:114274098-114274334 | *ZBTB20* | Hyper | 4.86E-05 | up | 4.86E-02 |
| chr7:110733611-110734196 | *LRRN3* | Hypo | 1.23E-06 | up | 7.92E-08 |
| chr3:98239609-98240661 | *CLDND1* | Hypo | 4.02E-07 | up | 1.68E-06 |
| chr22:39861489-39861702 | *MGAT3* | Hypo | 5.23E-05 | up | 9.36E-05 |
| chr10:330551-330864 | *DIP2C* | Hypo | 8.74E-05 | up | 9.76E-04 |
| chr12:95938919-95939513 | *USP44* | Hypo | 7.64E-05 | up | 1.76E-03 |
| chr2:207139319-207139585 | *ZDBF2* | Hypo | 7.32E-06 | up | 2.01E-03 |
| chr20:52062628-52063034 | *TSHZ2* | Hypo | 2.43E-05 | up | 4.68E-03 |
| chr7:105188266-105188675 | *RINT1* | Hypo | 7.54E-05 | up | 5.84E-03 |
| chr3:71538136-71538509 | *FOXP1* | Hypo | 1.63E-05 | up | 6.37E-03 |
| chr19:34787109-34787423 | *KIAA0355* | Hypo | 2.58E-05 | up | 6.72E-03 |
| chr3:183270227-183270709 | *KLHL6* | Hypo | 4.22E-05 | up | 8.33E-03 |
| chr13:28903787-28903962 | *FLT1* | Hypo | 7.02E-05 | up | 8.79E-03 |
| chr3:182970727-182971058 | *MCF2L2* | Hypo | 2.57E-07 | up | 1.20E-02 |
| chr5:372991-373651 | *AHRR* | Hypo | 4.19E-07 | up | 1.26E-02 |
| chr2:162929555-162930034 | *DPP4* | Hypo | 2.09E-05 | up | 1.32E-02 |
| chr1:25949058-25949336 | *MAN1C1* | Hypo | 9.94E-06 | up | 1.49E-02 |
| chr2:25286742-25287128 | *EFR3B* | Hypo | 3.54E-07 | up | 1.73E-02 |
| chr15:48483389-48483595 | *CTXN2* | Hypo | 2.75E-06 | up | 2.11E-02 |
| chr11:14664561-14664874 | *PDE3B* | Hypo | 6.37E-06 | up | 2.15E-02 |
| chr3:169757128-169757597 | *GPR160* | Hypo | 8.89E-05 | up | 2.49E-02 |
| chr15:48483389-48483595 | *SLC12A1* | Hypo | 2.75E-06 | up | 2.86E-02 |
| chr2:62421683-62421892 | *B3GNT2* | Hypo | 9.77E-06 | up | 3.56E-02 |
| chr19:19928614-19928997 | *ZNF506* | Hypo | 9.74E-05 | up | 3.73E-02 |
| chr18:3179372-3179957 | *MYOM1* | Hypo | 8.43E-07 | up | 3.80E-02 |
| chr12:94853449-94853898 | *CCDC41* | Hypo | 9.50E-07 | up | 3.88E-02 |
| chr14:99707876-99708459 | *BCL11B* | Hypo | 3.64E-06 | up | 4.77E-02 |
| chr17:2709716-2709887 | *RAP1GAP2* | Hypo | 5.05E-05 | down | 1.77E-04 |
| chr5:106907664-106908081 | *EFNA5* | Hypo | 2.21E-06 | down | 2.79E-04 |
| chr1:92946580-92947480 | *GFI1* | Hypo | 2.54E-05 | down | 1.21E-03 |
| chr1:32211861-32212281 | *BAI2* | Hypo | 2.26E-05 | down | 1.22E-03 |
| chr10:126749791-126750041 | *CTBP2* | Hypo | 3.46E-05 | down | 4.30E-03 |
| chr6:41889486-41889915 | *MED20* | Hypo | 4.24E-06 | down | 4.53E-03 |
| chr7:971754-972293 | *ADAP1* | Hypo | 7.53E-05 | down | 5.70E-03 |
| chr20:3204242-3204780 | *ITPA* | Hypo | 2.43E-05 | down | 5.83E-03 |
| chr7:148723197-148723699 | *PDIA4* | Hypo | 4.34E-05 | down | 7.82E-03 |
| chr4:1206731-1207090 | *CTBP1* | Hypo | 9.92E-05 | down | 1.13E-02 |
| chr17:7948581-7948972 | *ALOX15B* | Hypo | 5.96E-05 | down | 1.19E-02 |
| chr11:65656325-65656680 | *FIBP* | Hypo | 7.71E-07 | down | 1.21E-02 |
| chr21:45164636-45164914 | *PDXK* | Hypo | 4.01E-05 | down | 1.33E-02 |
| chr5:141255647-141256135 | *PCDH1* | Hypo | 2.06E-05 | down | 1.52E-02 |
| chr4:1858667-1859196 | *LETM1* | Hypo | 3.89E-05 | down | 1.80E-02 |
| chr9:128003249-128003712 | *HSPA5* | Hypo | 1.57E-05 | down | 1.85E-02 |
| chr3:47267209-47267717 | *KIF9* | Hypo | 5.43E-05 | down | 2.07E-02 |
| chr16:2208158-2208581 | *TRAF7* | Hypo | 5.77E-05 | down | 2.29E-02 |
| chr8:38835786-38836311  chr8:38831191-38831593 | *HTRA4* | Hyper  Hypo | 4.99E-05  6.30E-05 | down | 2.41E-02 |
| chr20:48763229-48763719 | *TMEM189* | Hypo | 3.12E-06 | down | 2.52E-02 |
| chr1:230248356-230248569 | *GALNT2* | Hypo | 8.04E-05 | down | 2.59E-02 |
| chr22:45066532-45066993 | *PRR5* | Hypo | 3.03E-05 | down | 2.65E-02 |
| chr12:111882672-111882996 | *SH2B3* | Hypo | 1.04E-05 | down | 2.82E-02 |
| chr22:37544494-37544856 | *IL2RB* | Hypo | 9.21E-05 | down | 2.84E-02 |
| chr19:39972336-39972578 | *TIMM50* | Hypo | 3.08E-05 | down | 3.03E-02 |
| chr1:20985181-20985505 | *DDOST* | Hypo | 4.92E-08 | down | 3.49E-02 |
| chr16:88839300-88839640 | *PIEZO1* | Hypo | 7.75E-05 | down | 3.69E-02 |
| chr6:41889486-41889915 | *BYSL* | Hypo | 4.24E-06 | down | 3.76E-02 |
| chr3:45138569-45138755 | *CDCP1* | Hypo | 4.20E-05 | down | 3.79E-02 |
| chr5:140888112-140888524 | *PCDHGC5* | Hypo | 7.25E-05 | down | 4.28E-02 |
| chr2:220419304-220419636 | *OBSL1* | Hypo | 5.13E-06 | down | 4.69E-02 |

Table S11. KEGG pathway enrichment analysis of common genes in methylation and mRNA datasets

| ID | Description | P value | Gene ID |
| --- | --- | --- | --- |
| hsa04144 | Endocytosis | 3.80E-03 | *IL2RA/ZFYVE9/AGAP1/LDLR/IL2RB/RAB35/AGAP3* |
| hsa04974 | Protein digestion and absorption | 6.37E-03 | *SLC16A10/COL4A3/DPP4/COL13A1* |
| hsa00510 | N-Glycan biosynthesis | 8.00E-03 | *MGAT3/MAN1C1/DDOST* |
| hsa04141 | Protein processing in endoplasmic reticulum | 1.15E-02 | *SIL1/PDIA4/MAN1C1/HSPA5/DDOST* |
| hsa05150 | Staphylococcus aureus infection | 1.84E-02 | *FCGR3A/CFH/FCGR2C* |
| hsa04380 | Osteoclast differentiation | 2.11E-02 | *FCGR3A/FCGR2C/MAP2K6/AKT3* |
| hsa05220 | Chronic myeloid leukemia | 2.47E-02 | *CTBP2/CTBP1/AKT3* |
| hsa04151 | PI3K-Akt signaling pathway | 2.60E-02 | *IL2RA/EFNA5/FLT1/COL4A3/C8orf44-SGK3/IL2RB/AKT3* |
| hsa04010 | MAPK signaling pathway | 3.43E-02 | *EFNA5/CACNB4/RASGRF2/FLT1/MAP2K6/AKT3* |
| hsa05235 | PD-L1 expression and PD-1 checkpoint pathway in cancer | 3.69E-02 | *ZAP70/MAP2K6/AKT3* |
| hsa04658 | Th1 and Th2 cell differentiation | 4.02E-02 | *IL2RA/IL2RB/ZAP70* |
| hsa04014 | Ras signaling pathway | 4.22E-02 | *EFNA5/RASGRF2/FLT1/ZAP70/AKT3* |
| hsa04659 | Th17 cell differentiation | 5.82E-02 | *IL2RA/IL2RB/ZAP70* |

Table S12. Difference in blood cytokine between smokers and nonsmokers

| Cytokine | β (95% CI) | P-value |
| --- | --- | --- |
| VEGF | 0.26 (0.02, 0.50) | 0.03 |
| FGF basic | 0.17 (-0.01, 0.34) | 0.04 |
| IL-1β | 0.26 (-0.20, 0.72) | 0.25 |
| IL-1ra | 0.25 (-0.12, 0.62) | 0.18 |
| IL-2 | 0.03 (-0.09, 0.15) | 0.66 |
| IL-4 | 0.24 (-0.01, 0.49) | 0.06 |
| IL-5 | 0.07 (-0.09, 0.24) | 0.37 |
| IL-6 | 0.08 (-0.13, 0.28) | 0.45 |
| IL-7 | 0.19 (-0.03, 0.48) | 0.08 |
| IL-8 | 0.64 (-0.87, 2.15) | 0.38 |
| IL-9 | 0.23 (-0.03, 0.46) | 0.08 |
| IL-10 | 0.02 (-0.19, 0.15) | 0.83 |
| IL-12 | 0.09 (-0.07, 0.25) | 0.24 |
| IL-13 | 0.31 (-0.35, 0.98) | 0.33 |
| IL-15 | 0.15 (-0.11, 0.41) | 0.23 |
| IL-17 | 0.17 (-0.04, 0.37) | 0.11 |
| Eotaxin | 0.70 (-0.09, 1.45) | 0.79 |
| G-CSF | 0.12 (-0.21, 0.45) | 0.45 |
| GM-CSF | 0.12 (-0.15, 0.39) | 0.36 |
| IFN-γ | 0.23 (-0.08, 0.54) | 0.14 |
| IP-10 | 0.06 (-0.74, 0.86) | 0.88 |
| MCP-1 | 0.25 (-0.49, 0.99) | 0.49 |
| MIP-1α | 0.58 (-1.00, 2.16) | 0.45 |
| PDGF-BB | 1.49 (-0.32, 3.30) | 0.10 |
| MIP-1β | 0.33 (-0.24, 0.91) | 0.24 |
| RANTES | 0.50 (-0.34, 1.34) | 0.22 |
| TNF-α | 0.18 (-0.04, 0.41) | 0.10 |
